# Consensus Statement on Vitamin D Status Assessment and Supplementation: Whys, Whens, and Hows

**DOI:** 10.1210/endrev/bnae009

**Published:** 2024-04-27

**Authors:** Andrea Giustina, John P Bilezikian, Robert A Adler, Giuseppe Banfi, Daniel D Bikle, Neil C Binkley, Jens Bollerslev, Roger Bouillon, Maria Luisa Brandi, Felipe F Casanueva, Luigi di Filippo, Lorenzo M Donini, Peter R Ebeling, Ghada El-Hajj Fuleihan, Angelo Fassio, Stefano Frara, Glenville Jones, Claudio Marcocci, Adrian R Martineau, Salvatore Minisola, Nicola Napoli, Massimo Procopio, René Rizzoli, Anne L Schafer, Christopher T Sempos, Fabio Massimo Ulivieri, Jyrki K Virtanen

**Affiliations:** Institute of Endocrine and Metabolic Sciences, San Raffaele Vita-Salute University and IRCCS Hospital, Milan 20132, Italy; Department of Medicine, Vagelos College of Physicians and Surgeons, New York, NY 10032, USA; Richmond Veterans Affairs Medical Center and Virginia Commonwealth University, Richmond, VA 23284, USA; IRCCS Galeazzi Sant’Ambrogio Hospital, Milano 20161, Italy; San Raffaele Vita–Salute University, Milan 20132, Italy; Department of Medicine, University of California and San Francisco Veterans Affairs Health Center, San Francisco, CA 94121-1545, USA; Department of Endocrinology, University of California and San Francisco Veterans Affairs Health Center, San Francisco, CA 94121-1545, USA; School of Medicine and Public Health, University of Wisconsin–Madison, Madison, WI 53726, USA; Faculty of Medicine, University of Oslo, Oslo 0313, Norway; Laboratory of Clinical and Experimental Endocrinology, Department of Chronic Diseases, Metabolism and Ageing, KU Leuven, 3000 Leuven, Belgium; Italian Foundation for the Research on Bone Diseases (F.I.R.M.O.), Florence 50129, Italy; Department of Medicine, Instituto de Investigación Sanitaria (IDIS), Complejo Hospitalario Universitario and CIBER de Fisiopatologia de la Obesidad y Nutricion (CIBERobn), Santiago de Compostela University, Santiago de Compostela 15706, Spain; Institute of Endocrine and Metabolic Sciences, San Raffaele Vita-Salute University and IRCCS Hospital, Milan 20132, Italy; Department of Experimental Medicine, Sapienza University, Rome 00161, Italy; Department of Medicine, School of Clinical Sciences, Monash University, Clayton 3168, Australia; Calcium Metabolism and Osteoporosis Program, WHO CC for Metabolic Bone Disorders, Division of Endocrinology, American University of Beirut, Beirut 1107 2020, Lebanon; Rheumatology Unit, University of Verona, Verona 37129, Italy; Institute of Endocrine and Metabolic Sciences, San Raffaele Vita-Salute University and IRCCS Hospital, Milan 20132, Italy; Department of Biomedical and Molecular Sciences, Queen's University, Kingston, Ontario, ON K7L 3N6, Canada; Department of Clinical and Experimental Medicine, University of Pisa, Pisa 56126, Italy; Faculty of Medicine and Dentistry, Queen Mary University of London, London E1 4NS, UK; Department of Clinical, Internal, Anesthesiologic and Cardiovascular Sciences, Sapienza University of Rome, Rome 00161, Italy; Unit of Endocrinology and Diabetes Campus Bio-Medico, University of Rome, Rome 00128, Italy; Division of Endocrinology, Diabetology and Metabolic Diseases, “Molinette” Hospital, University of Turin, Turin 10126, Italy; Geneva University Hospitals and Faculty of Medicine, Geneva 1205, Switzerland; Department of Medicine, University of California and San Francisco Veterans Affairs Health Center, San Francisco, CA 94121-1545, USA; Vitamin D Standardization Program (VDSP), Havre de Grace, MD 21078, USA; Institute of Endocrine and Metabolic Sciences, San Raffaele Vita-Salute University and IRCCS Hospital, Milan 20132, Italy; Institute of Public Health and Clinical Nutrition, University of Eastern Finland, Kuopio FI-70211, Finland

**Keywords:** vitamin D, cholecalciferol, calcitriol, calcifediol, vitamin D assay, Vitamin D Standardization Program (VDSP)

## Abstract

The 6th International Conference, “Controversies in Vitamin D,” was convened to discuss controversial topics, such as vitamin D metabolism, assessment, actions, and supplementation. Novel insights into vitamin D mechanisms of action suggest links with conditions that do not depend only on reduced solar exposure or diet intake and that can be detected with distinctive noncanonical vitamin D metabolites. Optimal 25-hydroxyvitamin D (25(OH)D) levels remain debated. Varying recommendations from different societies arise from evaluating different clinical or public health approaches. The lack of assay standardization also poses challenges in interpreting data from available studies, hindering rational data pooling and meta-analyses. Beyond the well-known skeletal features, interest in vitamin D's extraskeletal effects has led to clinical trials on cancer, cardiovascular risk, respiratory effects, autoimmune diseases, diabetes, and mortality. The initial negative results are likely due to enrollment of vitamin D-replete individuals. Subsequent post hoc analyses have suggested, nevertheless, potential benefits in reducing cancer incidence, autoimmune diseases, cardiovascular events, and diabetes. Oral administration of vitamin D is the preferred route. Parenteral administration is reserved for specific clinical situations. Cholecalciferol is favored due to safety and minimal monitoring requirements. Calcifediol may be used in certain conditions, while calcitriol should be limited to specific disorders in which the active metabolite is not readily produced in vivo. Further studies are needed to investigate vitamin D effects in relation to the different recommended 25(OH)D levels and the efficacy of the different supplementary formulations in achieving biochemical and clinical outcomes within the multifaced skeletal and extraskeletal potential effects of vitamin D.

## Graphical Abstract

**Graphical Abstract:**
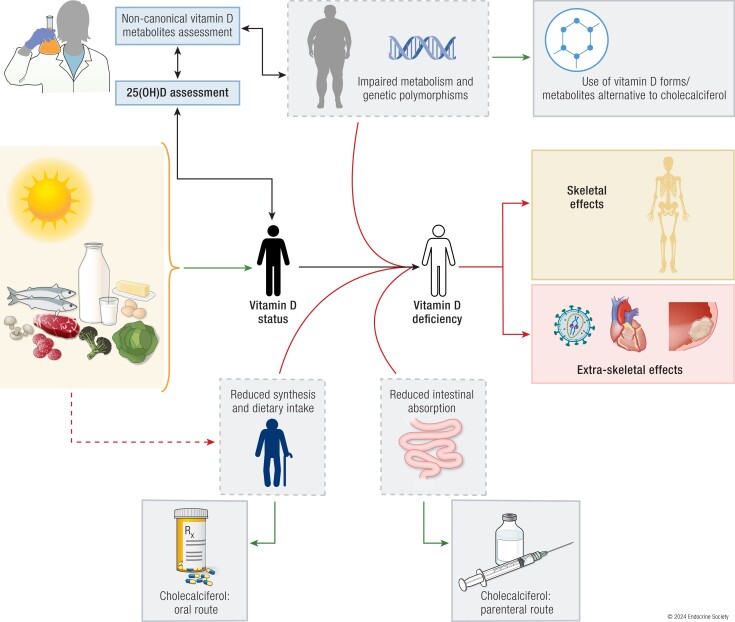

**Essential Points**

- Total serum 25-hydroxyvitamin D concentration is the accepted biomarker of vitamin D status, but assay methodology and standardization as well as desirable levels, which may vary according to the underlying condition, are still major issues
- Advances in knowledge about vitamin D have included its metabolism, identification of noncanonical metabolites, mechanisms of action, and genetic polymorphisms. These insights have added to our understanding of vitamin D's role in nutrition and in disease
- Vitamin D deficiency reduces intestinal calcium absorption leading to secondary hyperparathyroidism, bone loss, and increased risk of fractures in older adults. Meta-analyses of clinical trials show that vitamin D and calcium, together, decrease hip and other fractures in nursing home residents
- Post hoc analyses of recent mega trials on extraskeletal effects of vitamin D suggest a link between vitamin D status and immune system and development of type 2 diabetes mellitus. Cardiovascular events and mortality may be positively affected as well
- Daily vitamin D regimens seem to be the most efficient and beneficial strategy to improve vitamin D status but dosing schedules with longer intervals up to 4 weeks have been proposed to overcome low compliance with daily schedules
- Oral cholecalciferol (vitamin D_3_) remains the preferred form of vitamin D for supplementation, while other vitamin D analogues (eg, calcifediol, calcitriol, alfacalcidol) and parenteral administration should be used in specific conditions

The 6th International Conference “Controversies in Vitamin D” was held in Florence, Italy, September 21 to 24, 2022, as part of this series that started in 2017 (1-10). The objective of this conference, featuring international experts, was to review and discuss controversial topics regarding vitamin D. Before the event, participants reviewed the available literature on their assigned topic and presented their findings at the conference. After each presentation, open sessions enabled full discussion. On the last day of the conference, all participants completed their discussion and agreed on a menu for additional research. The 2 main topics addressed were recommendations on assessing vitamin D deficiency and vitamin D supplementation. This paper summarizes the findings on the “whys, whens, and hows” of these two topics.

## Vitamin D Metabolism and Mechanism of Action

### Metabolism

Vitamin D_3_ is produced in the skin from 7-dehydrocholesterol (7-DHC), while both vitamin D_2_ (ergocalciferol) and vitamin D_3_ (cholecalciferol) can be present in the diet. Vitamin D_2_ and D_3_ are hydroxylated first in the liver (and other tissues) to 25-hydroxyvitamin D (25(OH)D) and then in the kidney (and other tissues) to 1,25 dihydroxyvitamin D (1,25(OH)_2_D). Both 25(OH)D and 1,25(OH)_2_D are subsequently metabolized to their 24 (and for D_3_ 23) hydroxy forms 24,25(OH)_2_D_2/3_, 23,25(OH)_2_D_3_, and 1,24,25(OH)_3_D_2/3_ (or 1,23,25(OH)_3_D_3_). Like other steroid hormones, vitamin D is highly lipophilic and bound to protein carriers that help maintain stable serum levels. The half-life of serum 25(OH)D is 2 to 3 weeks, and that of the more water-soluble 1,25(OH)_2_D is approximately 5 to 8 hours. The majority of circulating 25(OH)D, including its metabolites, are bound tightly by vitamin D binding protein (DBP) and more loosely bound by albumin (4).

#### 7-Dehydrocholesterol reductase

Although the production of vitamin D from 7-DHC under the influence of sunlight (UVB) is a nonenzymatic step, the production of 7-DHC is not. Its synthesis in the skin is a step in the Kandutsch-Russell pathway. DHCR7 converts 7-DHC to cholesterol, so its activity dictates how much 7-DHC is available for vitamin D production. Inactivating mutations of DHCR7 result in Smith-Lemli-Opitz syndrome, a developmental disorder (11). These patients suffer primarily from the consequences of too little cholesterol, steroids, or bile acids, but they appear to be more sensitive to UVB light and may present with higher serum 25(OH)D concentrations than normal individuals. The regulation of DHCR7 is incompletely understood. Cholesterol and vitamin D (but not 1,25(OH)_2_D) increase proteasomal degradation of DHCR7, leading to increased vitamin D production. AMPK (adenosine monophosphate–activated protein kinase C), a key sensor and regulator of cellular energy homeostasis and protein kinase A are potent inhibitors of DHCR7 (12).

#### 25-Hydroxylases

The liver is the major source of 25(OH)D production from vitamin D. However, numerous enzymes within both mitochondria and microsomes have 25-hydroxylase activity. Initial studies suggested that CYP27A1, a mitochondrial enzyme with substantial homology to CYP27B1 and CYP24A1 (the 1α and 24-hydroxylases, respectively), was the major 25-hydroxylase. However, patients with inactivating mutations in this enzyme develop cerebrotendinous xanthomatosis with abnormal bile and cholesterol metabolism but not rickets (13). Current data support CYP2R1 as the major 25-hydroxylase, at least in the liver (and testes), where it resides in the microsomal compartment (13). When deleted in mice, serum 25(OH)D levels fall by over 50%, but not more. There is little effect on serum calcium and phosphate levels, suggesting that other enzymes with 25-hydroxylase activity compensate. Five functional mutations in CYP2R1 have been described so far. Although these mutations result in little or no 25-hydroxylase activity in vitro, individuals maintain normal or even high 1,25(OH)_2_D levels and, in some cases, respond both to vitamin D and 1α(OH)D with further increases in 1,25(OH)_2_D. As children, these individuals develop classical nutritional rickets responding to high doses of vitamin D or small doses of 25(OH)D; as adults, they tend to lose their need for vitamin D supplementation (14). Such data suggest that, as in the mouse, CYP2R1 could not be the only enzyme with 25-hydroxylase activity (14).

Previously, it had been thought that the 25-hydroxylation of vitamin D was primarily substrate dependent. However, recent evidence indicates that this is not the case. Roizen et al (15) found that the serum concentration of 25(OH)D, but not vitamin D, was decreased in mice fed a high-fat diet to induce obesity associated with decreased expression of CYP2R1 in the liver. Aatsinki et al (16) found that a high-fat diet that induced obesity and type 2 diabetes (T2D), as well as streptozotocin-induced type 1 diabetes, both decreased the hepatic messenger RNA and protein concentration of CYP2R1. Thus, the concept that the low levels of 25(OH)D in obesity and the limited response to vitamin D supplementation in these individuals are somehow related to increased storage of vitamin D in fat is still controversial (17) and needs further investigation.

#### CYP27B1—the 25-hydroxyvitamin D–1α-hydroxylase

Unlike the 25-hydroxylases, there is only a single 25(OH)D-1α-hydroxylase, CYP27B1. This enzyme is found in the mitochondrion along with CYP24A1. The kidney is the main source of circulating 1,25(OH)_2_D, but many tissues, including the epidermis and other epithelial tissues, bone, placenta, and immune system cells, also express CYP27B1. The product, 1,25(OH)_2_D, likely has paracrine or autocrine actions (18). Regulation of CYP27B1 in these extracellular sites differs from that in the kidney. In the kidney, CYP27B1 is regulated primarily by parathyroid hormone (PTH) and insulin-like growth factor-1, which stimulate it, as well as by fibroblast growth factor 23 (FGF23) and 1,25(OH)_2_D itself, which inhibit it. In nonrenal tissues cells, such as keratinocytes and macrophages, cytokines, such as, interferon-gamma (IFN-γ), tumor growth factor alpha (TNFα), and transforming growth factor beta1 (TGFβ1) are the major inducers of CYP27B1. In peripheral blood mononuclear cells, interleukin (IL)-1, IL-2, and IL-15 also stimulate CYP27B1 activity, whereas IL-4 is suppressive (19-21). Thus, the induction of CYP27B1 in these extrarenal tissues is by cytokines, and the failure of CYP27B1 in these tissues to respond to the increased circulating levels of 1,25(OH)_2_D and calcium account for the hypercalcemia often found in granulomatous diseases, such as sarcoidosis and lymphomas (22). Mutations in CYP27B1 cause a disease known as pseudovitamin D–deficiency rickets or type 1A vitamin D-dependent rickets (23); both the renal and extrarenal CYP27B1 have the same sequence, but their differences in regulation occur because of differences in tissue-specific multicomponent control modules within the regulatory regions of the gene.

#### CYP24A1 and CYP3A—the 25-hydroxyvitamin D–24(23) hydroxylases

These are the catabolic enzymes of vitamin D metabolism, with both 25(OH)D and 1,25(OH)_2_D as their substrates (24-26). CYP24A1 is the dominant 24-hydroxylase in most tissues, but CYP3A4 likely plays a role in the liver and intestine, where it is highly expressed. Both enzymes have 24-hydroxylase and 23-hydroxylase activity, although the relative proportions of 24-hydroxylase and 23-hydroxylase activity for CYP24A1 are species specific. Both enzymes are induced by 1,25(OH)_2_D—and CYP24A1 is induced by 25(OH)D as well (27)—and the induction of CYP3A4 seems to be at least as great as that for CYP24A1 in the intestine. To label CYP24A1 as a purely catabolic enzyme in vitamin D metabolism would appear to be a misnomer. 1,24,25(OH)_3_D has a substantial affinity for the vitamin D receptor (VDR), with approximately 10% of 1,25(OH)_2_D biological activity. Moreover, a specific G protein–coupled membrane receptor for 24,25(OH)_2_D, Fam57B2, has been identified in bone and other tissues such as the skin, and through this receptor, 24,25(OH)_2_D was found to be involved in fracture repair (28). CYP24A1 is under the control of 1,25(OH)_2_D and FGF23 (both stimulatory) and calcium (29). 5α-Dihydrotestosterone, via the progesterone receptor, has also been reported to stimulate CYP24A1 (30). In humans, inactivating mutations in CYP24A1 are now recognized as a major cause of idiopathic infantile hypercalcemia, a syndrome marked by severe hypercalcemia, hypercalciuria, and nephrocalcinosis, decreased PTH, low 24,25(OH)_2_D, and inappropriately normal to high 1,25(OH)_2_D. Although initially identified in children (31), more recent case reports indicate that the diagnosis may not be made until adulthood, generally following a condition of increased 1,25(OH)_2_D production like pregnancy (32, 33). Such adults generally present with early-onset nephrolithiasis and/or nephrocalcinosis.

Importantly, CYP3A4 mutations or drug-induced excess CYP3A4 activity have recently been linked to vitamin D deficiency and vitamin D–dependent rickets type 3, with affected individuals demonstrating greatly accelerated inactivation of vitamin D metabolites. This represents a novel mechanism for vitamin D deficiency (34).

### Mechanism of Action

**Figure 1.**
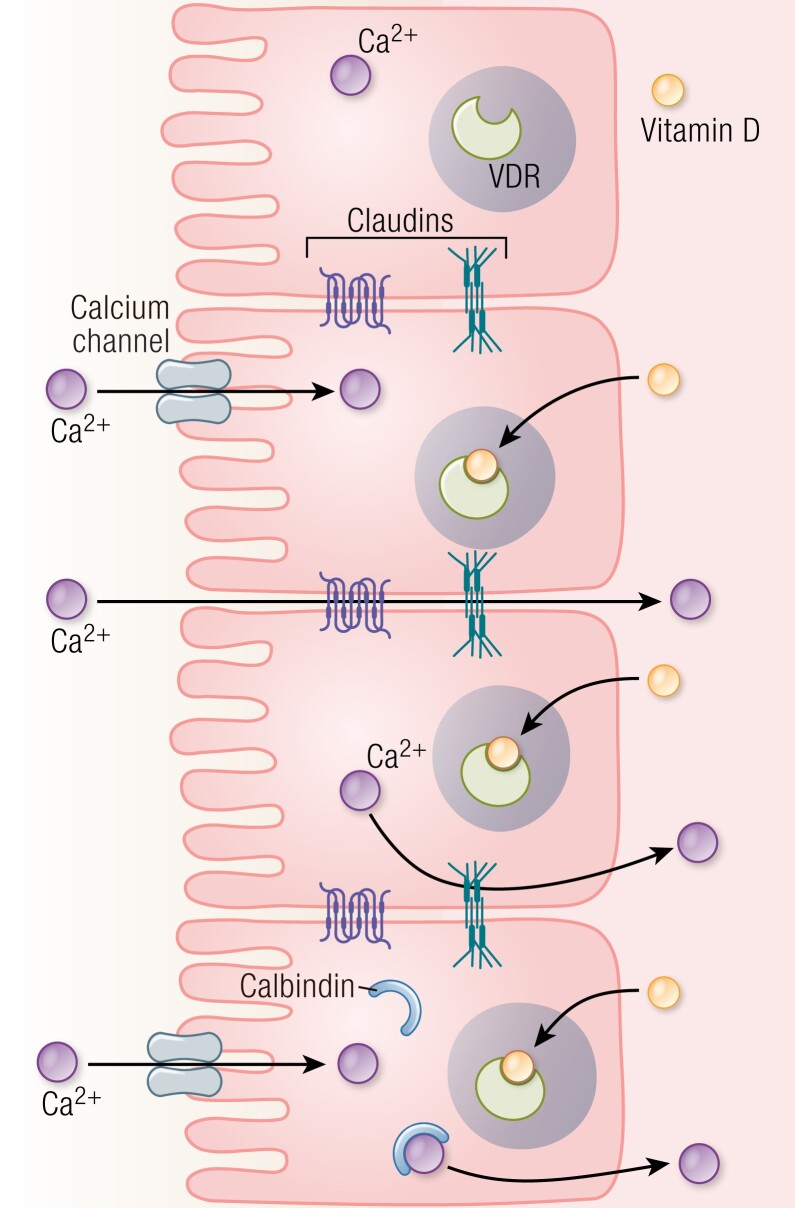
Three-step mechanism of intestinal calcium absorption by vitamin D. An important function of vitamin D is stimulating intestinal calcium absorption by increasing the expression of calcium-permeable claudins, apical membrane calcium channels, and calcium-binding protein calbindins. The extrusion of calcium is across the basolateral membrane. This process is especially enhanced when dietary calcium intake is low.

The VDR is critical for most of the actions of vitamin D, with 1,25(OH)_2_D as its major ligand. VDR is a transcription factor found in nearly all cells. Not surprisingly, vitamin D affects many cellular processes via the VDR, with one of the most important being the regulation of intestinal calcium absorption (Fig. 1) (4). In a recent ontology analysis (35), 11 031 putative VDR target genes were identified, of which 43% were involved with metabolism, 19% with cell and tissue morphology, 10% with cell junction and adhesion, 10% with differentiation and development, 9% with angiogenesis, and 5% with epithelial to mesenchymal transition. Furthermore, VDR can regulate various microRNAs (miRNAs) and long noncoding RNAs involving the expression of numerous proteins directly or indirectly. As a result of the appreciation that the VDR is so widespread along with the key vitamin D metabolizing enzymes such as CYP27B1 and CYP24A1, interest in understanding the role of vitamin D and the VDR in nonclassic as well as classic target tissues regulating calcium and phosphate homeostasis has been substantial. Although most of the actions of VDR involve its role as a transcription factor within the nucleus, the VDR has also been shown to have nongenomic actions via its location in the plasma membrane and perhaps even in mitochondria (4).

#### Regulation

The regulation of VDR expression is cell specific. For example, 1,25(OH)_2_D regulates VDR expression in bone cells but not in the intestine. Many factors in addition to 1,25(OH)_2_D regulate VDR expression, including growth factors, insulin, as well as PTH, glucocorticoids, estrogen, and retinoic acid, in some cases acting via a variety of transcription factors, such as AP-1, SP1, C/EBP, and CDX2, C/EBPβ, Runx2, cyclic adenosine monophosphate response element binding protein (CREBP), retinoic acid receptor (RAR), and glucocorticoid receptor (GR). Similarly, calcium upregulates VDR expression in the parathyroid gland, presumably through its calcium-sensing receptor. On the other hand, SNAIL 1 and 2 (SLUG) downregulate VDR expression in several cancer cell lines. MicroRNAs can regulate VDR levels, as exemplified by the binding of miR-125b, miR-298, and miR-27b to the 3′untranslated region to decrease VDR levels (4, 36).

#### Genomic actions

Carlberg (36) reported that the human genome contains more than 23 000 VDR binding sites, most of which are cell specific. Their locations varied with the duration of ligand exposure, and only some were readily identified with a specific gene. The VDR binding sites can be thousands of bases away from the transcription start site (TSS) of the genes they regulate, and genes generally have multiple VDR binding sites, the activity of which may vary in different cells and species. An informative example of how this might work in different cells is the regulation of the RANKL gene (*Tnfsf11*). This gene is regulated by PTH and 1,25(OH)_2_D in osteoblasts and by AP-1 factors, such as c-fos, in activated T cells (13). The Pike laboratory identified 7 VDR binding sites in RANKL up to 88 kb upstream of the TSS, of which the −75-kb site proved most active in the mouse gene (37, 38), whereas the proximal site was most active in the human gene (39). However, in activated T cells, 3 additional sites even further upstream of the TSS have been identified as sites of RANKL induction by c-fos (13).

A similar example can be found for *Cyp27b1*. This gene is negatively regulated by its product in the kidney but not in other tissues (40). The VDR binding sites are generally situated in a region with other transcription factors that may share regulation of that gene, potentially providing cell-specific gene regulation. For example, the VDR binding region of the *RANKL* gene contains several CREB sites responsible for the PTH regulation of this gene (41).

#### Coregulators and epigenetic changes regulating vitamin D receptor function

The sites of active transcription are marked by epigenetic changes both in the gene itself and the histones that regulate access of the transcriptional machinery to the gene. In humans and mice, 1,25(OH)_2_D regulates these epigenetic changes by affecting the binding of coregulators to the VDR, whether as coactivators with histone acetyltransferase activity (HAT) or as cosuppressors with histone deacetylase activity (HDAC). More than 250 published coregulators interact with nuclear hormone receptors. The best-studied coactivators with respect to the VDR are the steroid hormone receptor coactivators (SRC 1-3) and the Mediator complex. SRCs recruit HATs to the VDR. The Mediator complex does not contain HAT activity but binds directly to RNA polymerase II to help form the preinitiation complex along with basal transcription factors such as TFIIB and several TAT-binding proteins. These coactivators all bind to the AF2 domain of the VDR. On the other hand, corepressors, such as SMRT and NCoR complexes, have HDAC activity and bind to H3 to H5 in the absence of a ligand. In the presence of 1,25(OH)_2_D and the conformational change with H12, these corepressors are displaced, enabling the coactivators to bind to their sites on H12.

Hairless is a corepressor of VDR expressed primarily in the brain and skin. It binds to the central region of the ligand-binding domain of VDR, as does NCoR/SMRT. The role of hairless is complex in that it represses ligand-dependent VDR functions with respect to epidermal differentiation (42) but is required for ligand-independent VDR regulation of hair follicle cycling (43). In mice, *VDR* gene ablation elicits both rickets and hair loss, while point mutations specifically compromising either 1,25(OH)_2_D ligand or coactivator contacts in human VDR result in rickets without hair cycle disruption. On the other hand, loss-of-function mutations in human VDR results in disrupted VDR-DNA binding or VDR-RXR heterodimerization; this impaired corepressor activity on VDR-mediated transactivation, in part due to the attenuated interaction of hairless with HDACs, can result in clinical conditions, such as the rare autosomal recessive disease atrichia with papular lesions or alopecia universalis congenita (42-44). VDR interaction with its heterodimeric partner RXR is probably pivotal to hair cycling, as the conditional inactivation of RXRα in mouse skin results in alopecia resembling that in *VDR*-null mice. Similar to mutations in the *VDR*-encoding gene, mutations in the mammalian hairless gene result in congenital hair loss both in mice and humans. Remarkably, the hair loss phenotype caused by the mutated human *VDR* gene resembles the generalized atrichia caused by mutations in the hairless gene (44).

In summary, new insights into the regulation of vitamin D–related enzymes and the differential mechanism of action of VDR have demonstrated important links between metabolic disorders and vitamin D metabolism. A better understanding of how the VDR interacts with other transcription factors in a cell-specific fashion will provide a greater understanding of how the same molecule can have such different actions in many physiologic processes. In turn, more insights may lead to more nuanced and/or specific uses of vitamin D and its metabolites in clinical situations, as discussed next.

## Assessment of Vitamin D Status

**Figure 2.**
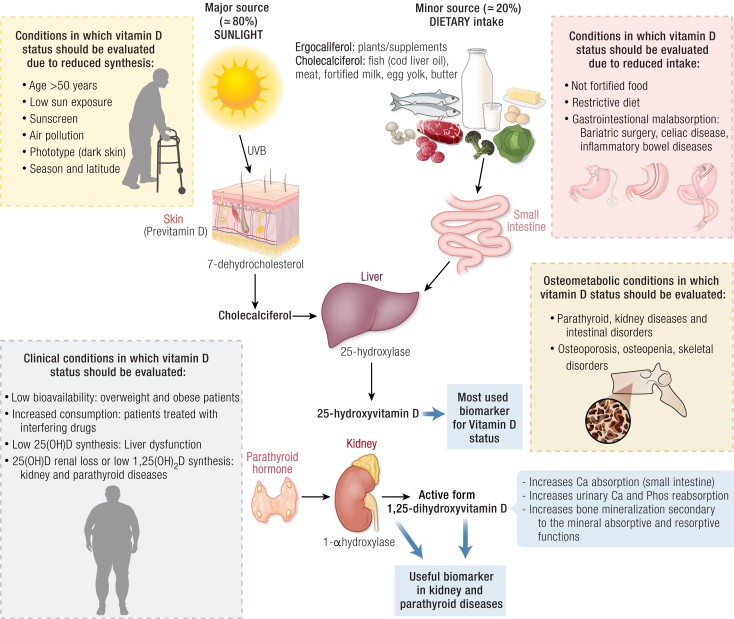
Overview of vitamin D metabolism. The figure shows metabolism of vitamin D in physiologic and deficient status, with specific reference to conditions in which vitamin D should be evaluated.

To date, total serum 25(OH)D, the sum of 25(OH)D_3_ and 25(OH)D_2_, is the accepted biomarker of vitamin D status (Fig. 2). Observational studies have indicated the beneficial effects of an optimal vitamin D status on various outcomes not directly associated with the classical target tissues for the hormone—the so-called pleiotropic effects (45). Based on these studies, mostly using traditional radioimmunoassay measurements, vitamin D guidelines issued by major organizations worldwide recommend optimal 25(OH)D levels to be in the range of 50 to 75 nmol/L (20-30 ng/mL) (46, 47). However, optimal levels are still debated for several reasons (48-50). Lack of assay standardization contributes to the problem, and initiatives should be implemented to overcome it (50, 51). In this perspective, the Endocrine Society (ES) has asked a task force to review its 2011 guidelines.

Differences in the suggested optimal serum 25(OH)D levels depend on several factors. It is essential to clarify what is meant by optimal 25(OH)D level, that is, for whom and for what, as it is essential to consider patients’ clinical profiles and the outcomes of interest. Many studies have been performed with a focus on osteoporosis and bone metabolism. Recently, several randomized controlled trials (RCTs) assessed potential pleiotropic effects of 25(OH)D, in general, with negative results (45, 52, 53). Another pivotal factor in deciding optimal 25(OH)D levels is the perspective used. For example, although the outcomes used to derive desirable 25(OH)D levels were similar for the Institute of Medicine (IOM) (47) and the 2011 ES guidelines (46), most studies included conclusions that differed. For example, in the case of osteomalacia, although the same study was used, conclusions differed. This is because the ES selected a cutoff above which no individual had osteomalacia (clinical perspective), whereas IOM chose a cutoff where 97.5% of the cohort did not have osteomalacia (public health perspective). Finally, when discussing vitamin D status assessment, it is also important to differentiate between screening, that is, a public health approach undertaken in the general populations, and testing, that is, targeted testing of high-risk individuals in the clinical setting.

Theoretically, obtaining a 25(OH)D level within an optimal window for the general population will necessarily result in overscreening and overtreatment of healthy individuals (48, 49). While general screening for 25(OH)D deficiency/insufficiency is not recommended, measurements could be performed in patients with several risk factors for severe deficiency or who are being evaluated for metabolic bone disease (46, 53-56). This recommendation may help to mitigate the dramatic increase in the number of 25(OH)D measurements and the associated economic burden (45, 52, 54, 56). Supporting this view, initiatives have been undertaken to reduce unnecessary 25(OH)D analyses in Australia (57, 58). The change in recommended testing criteria halved the number of measurements but paradoxically increased the number of unnecessary tests and decreased tests of patients at high risk of deficiency, with only a small improvement in the detection of deficiency (56).

### Screening and Testing for Vitamin D Status

#### Screening in the general population—public health approach

Levels of 25(OH)D in the general population vary considerably depending on several factors, including the season, latitude, cultural factors leading to reduced UVB light exposure, skin pigmentation, body mass index (BMI), sex, age, level of physical exercise, and food fortification with vitamin D or use of vitamin D supplements, even among otherwise comparable Western societies; moreover, genetic factors such as gene polymorphisms may have major effects on serum 25(OH)D according to twin studies and mendelian randomization (MR) reports (4, 48).

In considering when to test for vitamin D deficiency, it is well recognized that serum 25(OH)D levels vary by season. This is not surprising, given that most vitamin D is generated in the skin following UVB exposure with little vitamin D available from the average unfortified diet (59, 60). Given an observed drop in 25(OH)D levels between seasons, a higher target (∼75 nmol/L) may be required at the end of summer to allow for the anticipated 10 to 25 nmol/L drop during the winter months (61). For example, in a sunny country, such as Australia, the prevalence of vitamin D deficiency (<50 nmol/L) is as high as 36% during winter and as low as 14% in summer (62). In Lebanon, another sunny country, mean serum 25(OH)D levels were 12 to 15 nmol/L higher in summer to fall compared to winter (63). In this regard, measurement of serum 25(OH)D levels at the end of winter or in early spring would increase the detection of low 25(OH)D levels in the general population (61).

Regardless, in populations with a low prevalence of vitamin D deficiency, screening of the general population is not cost-effective, and the decision to assess an individual needs to be made using a risk stratification approach for having vitamin D deficiency. Prioritized screening for high-risk groups could be useful, given the potentially adverse effects of vitamin D deficiency on skeletal and overall health, particularly when the serum 25(OH)D levels are less than 30 nmol/L (<12 ng/mL). Boosted regression tree models have also been developed from RCT data to predict the serum 25(OH)D concentration (64). Several predictor variables of a deseasonalized serum 25(OH)D concentration less than 50 nmol/L have been identified from training and validation data sets in the D-Health trial. The 2 strongest predictors were ambient UV radiation and total intake of vitamin D. Other important predictors of mild vitamin D deficiency were time spent outdoors, alcohol consumption, BMI, quality of life measures, and physical activity. Thus, a lack of ambient UVB radiation and lack of vitamin D fortification of food or use of vitamin D supplements will probably result in a poor vitamin D status, particularly in individuals in whom other risk factors are also present.

In conclusion, screening for optimal vitamin D status in the general population should be avoided as it is not informative and has a considerable economic burden. Nevertheless, several characteristics and pathological conditions in the general population could place individuals at risk for severe deficits. These populations, which should be recognized, are considered in the next section.

#### Testing populations at risk of vitamin D deficiency—clinical approach

Measurement of 25(OH)D has been recommended in patients at risk for deficiency (46, 47) (Table 1 and Fig. 2). Thus, 25(OH)D is widely measured in many of these high-risk groups, for example, in older adults with decreased endogenous vitamin D production and prone to develop osteoporosis, in patients with parathyroid disorders and liver disease, and in patients with obesity (46, 47, 54-56, 65-71). Patients with class III obesity (BMI > 40) present with low levels of 25(OH)D for various reasons, including nutritional factors, psychological reasons leading to less sun exposure, decreased hepatic expression of CYP2R1, and sequestration of the vitamin in the excess adipose tissue (15, 72). Class III obesity may be addressed by bariatric surgery, which, by itself, may lead to malabsorption and, thereby, a further decrease of 25(OH)D levels, potentially followed by a secondary hyperparathyroidism (73). Other at-risk groups include those who are housebound, those working long hours indoors, dark-skinned individuals, patients with a chronic disease, those taking medications increasing vitamin D catabolism, etc (see Table 1). Paradoxically, listing situations where it may be reasonable to measure 25(OH)D accounts for most people. This would again result in overtesting with high costs for the health care system. Rather than testing in situations where it would be reasonable to, it would be better to test only in situations that actually warrant it. It comes down to the providers’ judgment in first recognizing these high-risk individuals and then deciding to confirm with a measurement of 25(OH)D. There is, in fact, little evidence for the scientific utility and cost-effectiveness of testing for 25(OH)D deficiency, even in some of these selected groups (53). For example, some guidelines have recommended against screening pregnant women for vitamin D deficiency because of uncertainty about the benefits of vitamin D supplementation for maternal and fetal outcomes (47, 74). However, a case can be made for optimizing vitamin D status in all pregnant or breastfeeding women and their offspring, given the reemerging public health concern of rickets in high-risk children (75) and potential benefits on future peak bone mass (76). Evidence of a relationship between low 25(OH)D and adverse maternal outcomes together with evidence that these adverse outcomes (eg, risk of preeclampsia, gestational diabetes, low birthweight, and the risk of severe postpartum hemorrhage) are reduced after vitamin D supplementation have also been shown (77, 78). Poor vitamin D status has also been associated with increased risk of low birth weight (79-81), increased risk of preterm birth (81, 82), and offspring's adverse anthropometric and neurodevelopmental outcomes (81), while supplementation or sufficient vitamin D status was found to be protective against the risk of low birth weight, preterm birth, and small for gestational age (80), and associated with improved offspring vitamin D sufficiency status, reduced fetal or neonatal mortality, and improved fetal and future linear growth (83, 84).

**Table 1.** Populations at risk of vitamin D deficiency according to a clinical approach

Older people
Housebound people Disabled people Institutionalized people
People working long hours indoors Office workers Factory or warehouse workers Taxi drivers Night-shift workers
People with dark skin
Low levels of physical activity
People with a debilitating/chronic disease Diabetes Chronic kidney disease Gastrointestinal malabsorptive syndromes Parathyroid disorders Liver diseases
Obesity—in particular those with highest levels of waist circumference
Patients after bariatric surgery
People taking medications increasing vitamin D catabolism: Phenobarbitone Carbamazepine Dexamethasone Rifampicin Nifedipine Spironolactone Ritonavir Cyproterone acetate
Babies of vitamin D-deficient mothers

Sources: (15, 46, 47, 54-56, 65-70, 72).

#### Screening and testing vitamin D status—conclusions

Screening the general population for vitamin D deficiency is very expensive and does not result in practical clinical benefits. 25(OH)D measurements have primarily been indicated in patients with musculoskeletal disorders, but the increased awareness of potential pleiotropic effects has widened the interest in screening, although without any definitive evidence.

The most important risk factors in the general population, as identified by recent studies, include low ambient UV radiation, low vitamin D intake, and gene polymorphisms. Their inclusion as primary risk factors in risk stratification approaches to assess vitamin D status will help effectively target 25(OH)D assessment in those most in need and at risk. Finally, further studies—including those with health economic measures—are warranted to best identify all the situations in which the assessment of vitamin D status is actually needed—or not.

### Methods: Assays, Thresholds, and Standardization

Accuracy and precision of vitamin D measurements are crucial to properly use the values obtained in biological fluids. The laboratory methods should be detailed in clinical trials, scientific papers, and even in the reports released to physicians and patients. The measurements could be obtained by either antibody-based methods (chemiluminescent or immunoenzymatic) or by liquid chromatography–mass spectrometry (LC-MS or LC-MS/MS), with the latter giving more consistent and accurate results; regardless, the reference material should also be indicated in the report (85). The laboratory should define the reference values considering the method used for analyzing the molecule(s). The unit of measure (molar or mass) should be clearly indicated. The mol/L unit should be preferred as the SI standard unit; alternatively, both mol/L and ng/mL should be reported. Moreover, the critical difference (or least significance change), or reference change values, that is, the value (percentage) testifying to a real modification of the molecule(s) concentration between 2 consecutive measurements, assayed with the same method, in the same patient, and, on the contrary, that the modification is not only dependent from analytical and biological variability (natural oscillation of values in individuals), should be known and properly considered (85).

Assay standardization remains a major challenge to interpreting data from various studies evaluating vitamin D and its metabolites and analogues. It should be a priority to enable rational pooling of data and implementation of meta-analyses relating specific vitamin D metabolites to various outcomes of interest (2). Indeed, it has been suggested that reporting standardized 25(OH)D results is required for funding and subsequent publication of vitamin D–related research data (51).

Mean bias between −5% and +5% is one of the two performance thresholds used to define a 25(OH)D assay as being standardized by the Vitamin D Standardization Program (VDSP) (2, 86). However, a flaw in that threshold is that an assay with a mean bias within that range may display enormous variability outside those limits when, in actuality, what is wanted is an assay with few measurements outside them (87).

Data from the VDSP's Vitamin D Standardization Certification Program conducted by the Centers for Disease Control and Prevention (CDC) in the November 2019 report—the last report before COVID-19—show the flaw in the mean bias threshold in assays certified by the CDC to be standardized (88). In the CDC's report, 20 immunoassays and 17 LC-MS/MS assays were certified as being standardized for serum total 25(OH)D measurement (Table 2).

**Table 2.** Mean individual samples pass rate for 40 serum samples by Centers for Disease Control–certified standardized laboratories by assay type

Assay type	No., certified	Individual samples pass rate, %			
		Mean, %	SD	Minimum	Maximum
Immunoassay	20	30*^a^*	12.5	8	68
LC-MS/MS	17	61*^a^*	14.0	38	88

CDC individual samples pass rate is the percentage of individual samples out of 40 provided that met the certification criteria of ±5% bias. This information was provided starting in February 2017. Data analyses by Prof Christopher Sempos.

Source: CDC (87).

Abbreviations: CDC, Centers for Disease Control and Prevention; LC-MS/MS, liquid chromatography–tandem mass spectrometry.

^*a*^ *t* = −7.2; *P* = .00001.

The mean individual samples pass rate for LC-MS/MS assays (61%) was 2 times higher than the rate for immunoassays (30%) (*t* = −7.2; *P* < .01). LC-MS/MS assays provided the highest mean value (mean = 61%); however, there was considerable overlap. The Fujirebio Lumpulse had the highest pass rate for an immunoassay: 68%. The Boditech Ichroma had a 65% pass rate, and 3 immunoassays had a 42% pass rate, namely the Abbott Architect, IDS CLIA, and Siemens Maglumi ones. The VDSP's definition of the mean bias threshold should thus be revised. The criteria we suggest for any revision are (1) consistent with the original guidelines, that is, not an abrupt change; (2) easily calculated and easily understood; (3) easy to operationalize; (4) easily modified to promote change over time; and (5) will promote competition among assay manufacturers.

Importantly, we also suggest that 25(OH)D assays continue to monitor their regular performance using an external quality assessment scheme that provides target reference values from a reference measurement procedure approved by the Joint Committee for Traceability in Laboratory Medicine provided by an LC-MS/MS standardized assay (eg, DEQAS, Charing Cross Hospital, London UK). Such a process emphasizes the importance of assay accuracy and, given that true concentration is available in such specimens, allows their use in retrospective standardization of 25(OH)D data (2, 89).

### Assessment of Other Vitamin D Forms and Main Metabolites

As discussed earlier, the vitamin D status assessment is based on the 25(OH)D serum level measurement. However, other forms of vitamin D such as free 25(OH)D, bioavailable 25(OH)D, DBP, or 1,25(OH)_2_D levels could be used as biomarkers of vitamin D repletion, defined by effect on classical and nonclassic vitamin D outcomes. As for 25(OH)D measurements, these tests would also need to be standardized to ensure accuracy and replicability.

For circulating 25(OH)D, it is estimated that approximately 85% to 90% is bound by DBP and 10% to 15% by albumin; therefore, free 25(OH)D levels are estimated to be less than 1% of the total and can vary according to DBP and albumin polymorphisms and binding affinity (90-92). The free and not the total 25(OH)D concentration in cell cultures affects a biological response. While this is harder to assess in vivo, some tissues with the megalin/cubilin complex, like the kidney and parathyroid gland, can take up the vitamin D metabolites bound to DBP (93). Nonetheless, free 25(OH)D may be highly relevant to local intracellular (eg, osteoblasts, renal cells, muscle cells) synthesis of 1,25(OH)_2_D, which can behave in a paracrine and autocrine fashion (4). In normal populations, total and free 25(OH)D, as well as free and calculated 25(OH)D, are correlated (∼60%-70% in healthy individuals), and there is no clear evidence for a need to measure free metabolites in healthy individuals and many clinical settings (92-97). However, this may not hold true in conditions affecting DBP such as pregnancy, cirrhosis, acute illness, conditions that may affect the affinity of DBP or albumin to its ligands, and even in aging nursing home residents (4, 69, 94), for whom the free concentration is a better assessment than the total.

Measurement of 1,25(OH)_2_D may contribute to the diagnosis of conditions with low calcitriol levels, such as 1α-hydroxylase deficiency, or those associated with high 1,25(OH)_2_D levels, such as hereditary vitamin D–resistant rickets, granulomatous conditions (sarcoidosis and tuberculosis), and the hypophosphatemic syndromes (4, 69).

Available evidence to date is rather limited to determine whether free or bioavailable 25(OH)D or 1,25(OH)_2_D is the better biomarker of 25(OH)D availability to local tissues and of its effect on target organs in special situations. The extremely low serum concentrations of 25(OH)D and 1,25(OH)2D found in mice and humans with genetic absence of DBP without implications on calcium homeostasis is the best argument for the free “vitamin D” hypothesis.

### Assessment of Other Metabolites

Improvements in LC-MS/MS have triggered a revolution in small-molecule clinical chemistry, particularly the analysis of steroid hormones. The additional sensitivity and selectivity provided by the recently emerged LC-MS/MS techniques make it now feasible to measure most of the circulating vitamin D metabolites of value to clinicians and physiologists in human and animal studies. A comprehensive analysis can now assay 8 metabolites simultaneously (cholecalciferol, 25(OH)D, 3-epi-25(OH)D, 24,25(OH)_2_D, 25,26(OH)_2_D, 1,25(OH)_2_D, 1,24,25(OH)_3_D, and 25(OH)D-26,23-lactone) by judicious use of liquid-liquid-extraction and immune-extraction steps. Besides specific clinical situations (98, 99), a few previous reports have also highlighted a potential role for vitamin D metabolites, in particular of the 24,25 to 25(OH)D ratio, in better-predicting fracture risk as compared to only 25(OH)D levels (100, 101).

#### Infantile hypercalcemia, type 1, caused by defects in CYP24A1

Despite the name of the disease, infantile hypercalcemia, type 1 affects individuals throughout life, usually causing nephrolithiasis. It is especially problematic in pregnant females due to the placental production of 1,25(OH)_2_D_3_, which cannot be efficiently metabolized. The utility of measuring 24-hydroxylated forms, particularly the 25(OH)D to 24,25(OH)_2_D ratio, has been established as a useful screening tool by groups worldwide. Ratios are elevated from 5 to 25 in normal individuals to more than 80 in infantile hypercalcemia–affected individuals (102). It is important to recognize that this same enzymatic defect can be identified in adults with unexplained 1,25(OH)_2_D-dependent hypercalcemia. These individuals present with hypercalcemia, hypercalciuria, kidney stones, and suppressed levels of PTH.

#### Other hypercalcemias

Many causes of hypercalcemia can be distinguished by their distinctive pattern of vitamin D metabolites. Kaufmann et al (98) identified several patient groups by studying the vitamin D metabolome. These include patients with Williams syndrome exhibiting an elevated level of 25(OH)D-26,23-lactone, a stable metabolite with high affinity for DBP; patients with hypervitaminosis D taking toxic doses of vitamin D exhibiting very high 25(OH)D but suppressed 1,25(OH)_2_D, and where several other vitamin D metabolites may contribute.

#### Chronic kidney disease

Many studies have documented a fall in serum 25(OH)D and 1,25(OH)_2_D with a decline in renal function. Studies of the vitamin D metabolome over the 5 stages of chronic kidney disease (CKD) have revealed that the same phenomenon also applies to 24,25(OH)_2_D. Patients and animal models with experimental kidney disease both show changes in the levels of 24,25(OH)_2_D_3_ and 1,24,25(OH)_3_D_3_ with changes in glomerular filtration rates (103). The clinical consequences of these changes remain to be elucidated.

#### Routine documentation of vitamin D metabolites in randomized controlled trials

In most recent large RCTs, participants were monitored only for health effects and serum 25(OH)D levels. One study—the so-called Calgary study (or JAMA study) (104)—used doses of up to 10 000 international units (IU) of vitamin D/day, monitored only 25(OH)D, and reported deleterious effects of the vitamin D on bone mineral density (BMD) (BMD was assessed with high-resolution peripheral quantitative computed tomography and not with dual-energy x-ray absorptiometry). Although this JAMA study reported only 25(OH)D data (105), by reanalyzing the serum from participants in the study for the full vitamin D metabolome including 1,25(OH)_2_D_3_, 24,25(OH)_2_D_3_, and 1,24,25(OH)_3_D_3_, Burt and colleagues (105) found that several vitamin D metabolites, including 1,24,25(OH)_3_D_3_ but not 1,25(OH)_2_D_3_, were elevated in individuals given the 10,000 IU of vitamin D/day dose, a fact that could explain the bone loss observed at high supplementation rates.

In conclusion, the study of a wider array of vitamin D metabolites provides insight into a limited number of diseases. It can potentially improve understanding of vitamin D status and its relationship to multiple diseases.

## Clinical Outcomes of Vitamin D Deficiency

### Skeletal Outcomes

**Figure 3.**
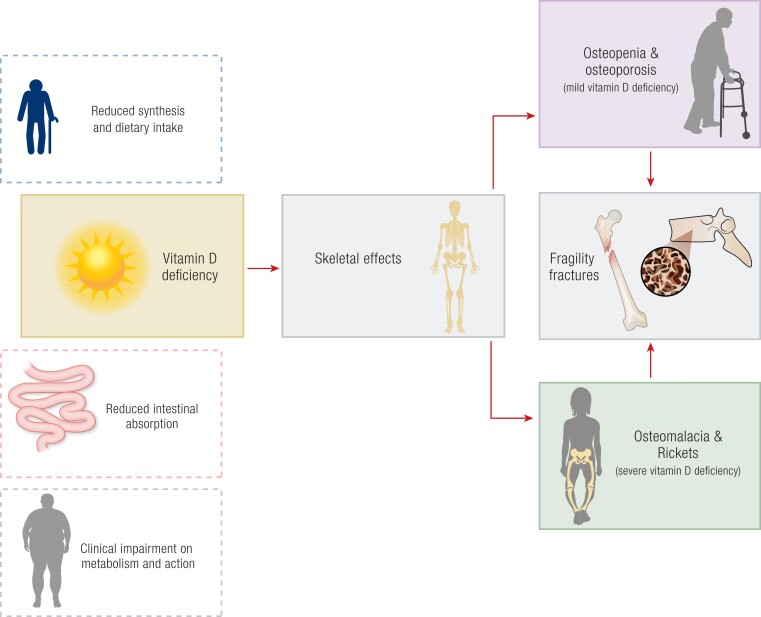
Skeletal effects of vitamin D deficiency. A deficient vitamin D status can cause impairments in the skeletal system such as osteopenia, osteoporosis, osteomalacia, and rickets, resulting in high risk for fragility fractures. Clear boxes with dashed outlines refer to the risk factors for vitamin D deficiency; dark boxes refer to the negative skeletal effects of vitamin D deficiency.

Skeletal outcomes of vitamin D deficiency are summarized in Fig. 3. Vitamin D deficiency leads to a decrease in intestinal absorption of calcium and phosphate. Other biochemical abnormalities, such as hypocalcemia, hypophosphatemia, and an increase in alkaline phosphatase, become apparent when serum 25(OH)D concentrations are lower than 25 nmol/L (106). In milder forms of vitamin D deficiency, the lower calcium concentration causes secondary hyperparathyroidism, which increases the conversion of 25(OH)D into 1,25(OH)_2_D, increasing calcium absorption and correcting serum calcium (4, 107). Secondary hyperparathyroidism causes an increase in bone turnover, with relatively higher bone resorption at cortical sites (107-112). More severe longstanding vitamin D deficiency causes a decrease in the mineralization of newly formed osteoid tissue. This is visible in bone biopsies as an increase in osteoid surface and volume and increased thickness of osteoid seams, leading to the clinical picture of osteomalacia (4, 107). Vitamin D deficiency and related secondary hyperparathyroidism cause bone loss and fractures in older adults. The incidence of hip fractures attributable to vitamin D deficiency has been estimated at 5% to 10% (113). Meta-analyses of clinical trials with vitamin D and calcium have demonstrated a decrease in hip and other fractures of around 10% in nursing home residents, whereas vitamin D alone was not effective (113-115). In these studies, baseline mean serum 25(OH)D after cross-calibration was found to be very low—namely less than 25 nmol/L—as was the calcium intake (116). As almost all effective trials used a calcium supplement in addition to vitamin D, the effect on BMD of vitamin D supplements alone is difficult to determine, but it is considered to be less than 1% (113), and high doses may even be harmful when administered to vitamin D–replete individuals (104). Recent RCTs such as the ViDA, VITAL, and D-Health studies do not show skeletal benefits for mostly vitamin D–replete adults and older individuals; for example, in the VITAL trial, cholecalciferol supplementation did not result in a significantly lower risk of fractures (total, nonvertebral, and hip fractures) than placebo among generally healthy midlife and older adults not selected for vitamin D deficiency, low bone mass, or osteoporosis (117, 118). In the D-Health study, large bolus monthly doses (60 000 IU) resulted in no increase nor decrease in fracture risk overall. However, the hazard ratio appeared to decrease with increasing follow-up time (119). Interestingly, in a recent retrospective longitudinal study (120), the use of cholecalciferol was associated with reduced incidence of morphometric vertebral fractures in high skeletal risk, such as acromegaly (121).

In a recent umbrella review of meta-analyses of vitamin D RCTs, the only consistent significant findings were for calcium and vitamin D, and not vitamin D alone, in reducing the risk of hip fractures by 16% to 39%, in 8 of 13 meta-analyses, and of any fracture, by 5% to 26%, in 8 of 14 meta-analyses. These findings were driven by events in institutionalized older and frailer individuals (122).

In children, the lack of calcium and phosphate causes the expansion of the epiphyseal growth plates due to decreased apoptosis of the hypertrophic chondrocytes, clinically visible as thickening near the joints and radiologically as widening of the growth plates (4). The weaker bone leads to typical deformities, such as knock knees (genua valga) and bowlegs (genua vara). The occurrence of rickets is mainly restricted to the Middle East and some countries in Asia, such as Mongolia and parts of China and India (123, 124), while it is also observed in immigrants and refugees in other countries (125).

### Extraskeletal Outcomes

**Figure 4.**
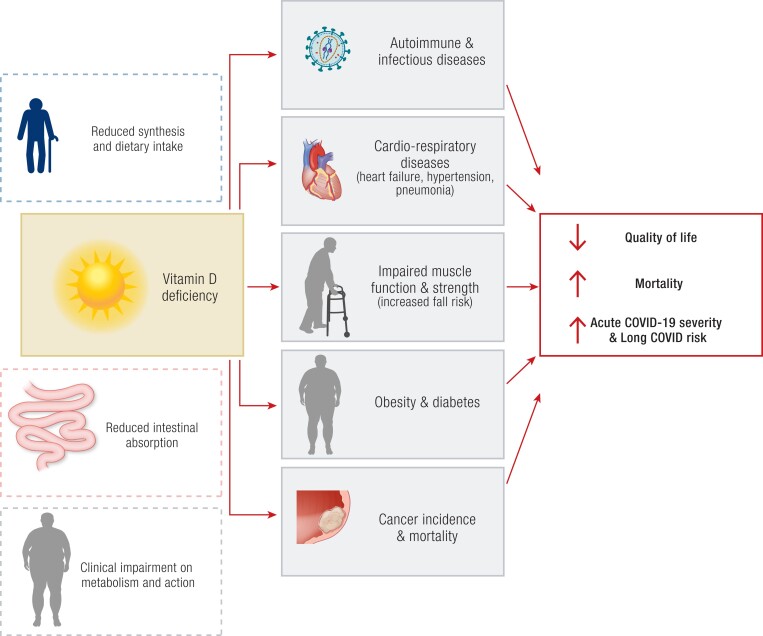
Putative extraskeletal effects of vitamin D deficiency and their implication in human health. A deficient vitamin D status is associated with several extraskeletal effects. These include increased risk of diabetes and autoimmune, infectious, cardiovascular, and respiratory diseases, as well as increase in cancer incidence and mortality. Such impairments result in lower quality of life and higher mortality, and can even increase acute COVID-19 severity and long COVID risk. Clear boxes with dashed outlines refer to the risk factors for vitamin D deficiency; dark boxes refer to the negative extra-skeletal effects of vitamin D deficiency.

Putative extraskeletal outcomes of vitamin D deficiency are summarized in Fig. 4. There are many preclinical data on the extraskeletal effects of the vitamin D endocrine system, including gene regulation, cellular function, and in vivo animal studies. Indeed, about 3% of the mammalian genome is under some control of vitamin D, and most cells express VDR or can synthesize the active hormone 1,25(OH)_2_D locally. Observational data largely align with these data as poor vitamin D status is associated with many human diseases (4). To complete the observational data, many large-scale trials that evaluated the effects of vitamin D supplementation on several extraskeletal health outcomes have been carried out recently, including the large VITAL (USA) (118, 126-129) and D-Health (Australia) (64, 129, 130) studies, as well as the ViDA (New Zealand) (131-133), FIND (Finland) (134, 135), and the D2d (USA) (136, 137) trials (Table 3). Smaller scale studies, such as the Calgary and the DO-Health (Switzerland), provide additional data. Moreover, there are now good genetic data on the prediction of serum 25(OH)D, which resulted in about 100 MR studies (117).

**Table 3.** Characteristics and results of the most recent and largest randomized controlled trials on vitamin D supplementation

Study	Participants (n)	Age (mean ± SD), y	Sex (% of women)	Mean BMI	Ethnicity*^a^* (% White ethnicity)	Serum 25(OH)D, ng/mL		Dose used	Follow-up, y	Primary outcome(s)*^c^*	Conclusions and comments
						Baseline	Final*^b^*				
**VITAL (117, 125-128)**	25 871	67 ± 7	51	28	71	30.8 ± 10	42 ± 10	2000 IU/d + omega-3 1 g/d	5.3	Invasive cancers and major CV events	End point not met, but reduction in total cancer mortality when excluding first 1-2 y of follow-up
										Incidence of metastatic or fatal cancer	VD reduced metastatic or fatal cancers by 17%; strongest reduction in normal BMI
										Two or more falls and falls resulting in a doctor or hospital visit	End point not met
										All incident autoimmune diseases	VD reduced autoimmune diseases by 22%
										Incident total, nonvertebral, and hip fractures	End point not met; enrolled individuals were generally healthy and not selected for VD deficiency, low bone mass, or osteoporosis
**D-Health (64, 129, 130)**	21 315	69.3	46	28	96.5%	31 ± 10*^d^*	46 ± 12	60 000 IU/mo	5.7	All-cause mortality	End point not met; VD increased cancer risk when first 2 y of follow-up were excluded
										Risk of falling	End point not met; VD increased risk when BMI <25, but not when BMI ≥25
										Major CV events	End point not met; VD might reduce CV events (small absolute risk difference and CI consistent with null finding); VD reduced myocardial infarction by 19%
**ViDA (131, 132, 138)**	5110	66 ± 8	58	29 ± 5.1	83	27 ± 9*^e^*	54 ± 16	200 000 IU + 100 000 IU/mo	3.3	Incident CVD and death	End point not met (121); in one substudy, VD lowered central blood pressure in deficient participants
										Fractures and falls	End point not met
										Cancer incidence and mortality	End point not met; daily or weekly dosing for longer period may require further study
**FIND (133, 134)**	2495	685	43	27 ± 4	100	30 ± 7	40 ± 9 (1600 IU/d arm) 48 ± 9 (3200 IU/d arm)	1600 or 3200 IU/d	4.3	Incident major CVD and invasive cancer	End point not met; study failure possibly due to sufficient VD status in most participants at baseline
										Atrial fibrillation risk	VD reduced atrial fibrillation risk by 27%-32%
**D2d (135, 136)**	2423	60 ± 10	45	32 ± 5	67	28 ± 10	54 ± 15	4000 IU/d	2.5	T2D in adults with prediabetes	End point not met
										Development of T2D according to intratrial serum 25(OH)D level	VD resulting in 25(OH)D level ≥100 nmol/L reduces risk of T2D

Partly drafted with data from Bouillon et al (117).

Abbreviations: 25(OH)D, 25-hydroxyvitamin D; BMI, body mass index; CV, cardiovascular; CVD, cardiovascular disease; IU, international units; N.A., not available; T2D, type 2diabetes; VD, vitamin D.

^*a*^The VITAL and D2d studies included different American racial and/or ethnic groups including Black people and Hispanic people. The ViDA study included Asian people and a small number of indigenous Māori individuals.

^*b*^Final serum 25(OH)D concentration vitamin D-treated groups only.

^*c*^Primary outcome(s) refers both to main trial and subsequent analyses.

^*d*^Evaluated in placebo group during follow-up.

^*e*^Deseasonalized mean values.

#### Cancer

No effects of vitamin D supplementation on cancer risk were observed in the large VITAL and ViDA trials, nor the FIND trial using daily dosing in older participants, nor on cancer mortality in the D-Health study, which used monthly dosing—in line with prior trials and MR results (117, 130, 134). Based on several MR studies, small changes in vitamin D status are unlikely to affect cancer incidence (117). However, a subanalysis of the VITAL trial (although not corrected for multiple end point analysis) showed that vitamin D supplementation could have some minor benefits in individuals with normal BMI (128). In addition, several independent trials have suggested, in post hoc analysis, the potential benefits of vitamin D supplementation on cancer mortality, especially when the follow-up is longer than 4 years (139). A meta-analysis of RCTs suggested that vitamin D supplementation decreased cancer mortality (140); an updated version of this study specifically designed to examine whether results varied by daily vs infrequent large-bolus dosing and by whether the trial participants had obesity or not found that overall benefit of vitamin D supplementation is lost when all the studies are considered. However, when considering daily regimens, vitamin D supplementation reduced total cancer mortality and incidence in normal-weight individuals (141). Therefore, a link between vitamin D status and cancer incidence or mortality can be hypothesized, and supplementation might be effective only with daily dosages, especially in people with BMI within a normal range (117, 141).

#### Cardiovascular risk

Convergent evidence from MR studies and RCTs suggests that vitamin D supplementation does not decrease the risk of cardiovascular disease (CVD), especially in vitamin D–replete adults. This conclusion may also apply to those with vitamin D deficiency based on subgroup analyses of the ViDA and VITAL trials. However, both studies recruited very few participants with severe vitamin D deficiency (117), rendering these conclusions uncertain. These null findings were corroborated by a meta-analysis of 21 RCTs (142). Nonetheless, more recent findings might suggest some small benefits. A detailed analysis of the ViDA trial found some modest benefits on central (but not peripheral) blood pressure, but the implications of this observation are limited because of the small scale of this ViDA substudy (133). The FIND trial failed to note a reduction in the number of major CV events, which was one of the two primary end points (134); however, subsequent exploratory analyses revealed that high-dose vitamin D supplementation might result in benefits in atrial fibrillation prevention in older individuals, even in case of relatively high baseline 25(OH)D concentrations (135). In the D-Health trial, the overall rate of major CV—and especially the rate of myocardial infarction and coronary revascularization—was lower in the intervention group compared to the placebo group, although the absolute risk difference was small, and the CI was consistent with a null finding (hazard ratio 0.91; 95% CI, 0.81-1.01); moreover, the protective benefits could be higher in those taking CV drugs at baseline (138).

#### Respiratory effects

Vitamin D is known to influence the immune system. Most immune cells express the VDR and vitamin D metabolism–related enzymes; 1,25(OH)_2_D, in particular, induces innate antimicrobial effector mechanisms such as the antimicrobial peptides cathelicidin LL-37 and human beta-defensin 2 (4). Indeed, clinical data regarding the effects of adequate vitamin D status and supplementation on respiratory infections confirm, at least in part, its potential beneficial outcomes. Serum 25(OH)D levels of less than 25 nmol/L are associated (observationally and genetically) with an increased risk of bacterial pneumonia (143). Individual participant data from a meta-analysis of 25 trials showed a small but significant decrease in the incidence of acute respiratory infections in the vitamin D group compared with the control group when baseline vitamin D status was poor (<25 nmol/L) (144). A more recent, updated meta-analysis from the same group, including almost 50 RCTs, shows a protective but very small effect against respiratory infections following vitamin D supplementation with daily doses of 400 to 1000 IU; in contrast to their first meta-analysis, baseline vitamin D status did not modify the results in this more recent one (145).

As respiratory tract infections are common in children, some promising data are available also in this setting. Children with poor vitamin D status were reported to be more prone to developing respiratory infections, although a conclusive association between the severity of respiratory infections and low vitamin D levels was not clearly established (146). RCTs show that vitamin D supplementation can benefit infants, toddlers, and preschool children aged 0 to 5 years with a quicker recovery and fewer respiratory symptoms (147). Unfortunately, study heterogeneity in terms of design, vitamin D supplementation doses, and duration, along with participant characteristics, make it problematic to pool data and, thus, difficult to draw definitive conclusions (79, 147).

There is also consistent evidence for an association between low 25(OH)D levels and poor COVID-19 outcomes, although the evidence supporting a beneficial effect of vitamin D supplementation in decreasing the risk of COVID-19 complications is conflicting (8, 148-151). An MR study found no evidence that vitamin D is protective against SARS-CoV-2 infection or COVID-19 severity (152). However, a meta-analysis of several observational studies comprising almost 2 million adults suggests that inadequate vitamin D status increases susceptibility to COVID-19 and severe COVID-19, while the association with mortality was less robust. Of note, the included studies were at high risk of bias and heterogeneity, and the heterogeneity in RCTs precluded their meta-analysis (151). Furthermore, low 25(OH)D levels were also recently associated with an increased risk for long COVID occurrence (150). However, a phase 3 RCT found no effect of vitamin D supplementation on the risk of developing long COVID after an episode of COVID-19 (142). Also, deficient vitamin D status was recently reported to be associated with a reduced long-term immune response to the anti–COVID-19 vaccination (153).

Vitamin D supplementation also seems effective in safely and substantially reducing the rate of moderate/severe acute exacerbations of chronic obstructive pulmonary disease in patients with baseline 25(OH)D levels less than 25 nmol/L—but not in those with higher levels (154). A meta-analysis, conversely, found no role for vitamin D supplementation in improving expiratory lung function (155).

Regarding asthma, there are insufficient RCTs to evaluate the potential benefit of vitamin D or its hydroxylated metabolites in improving its control or reducing the risk of exacerbations. However, as individuals with baseline 25(OH)D levels less than 25 nmol/L and those with severe asthma were poorly represented, and since one study investigating the effects of calcidiol yielded positive results, further studies are warranted in these populations and settings (156).

#### Autoimmune diseases

Conversely, from the innate immune system, the adaptive immune system is downregulated by 1,25(OH)_2_D in animal models. Thus, vitamin D deficiency might predispose to autoimmune diseases. Observational studies have suggested this effect might apply to humans (4).

The VITAL RCT showed that vitamin D supplementation decreased the risk of autoimmune diseases, especially rheumatoid arthritis and polymyalgia rheumatica, and at least 8 large MR studies all agree that genetically predicted lower 25(OH)D levels increased the risk of developing multiple sclerosis either during adolescence or adulthood (117, 129). In any case, the low number of intervention studies so far conducted does not allow clarification of the relationship between vitamin D and autoimmune diseases. However, these studies to date seem promising.

#### Diabetes

Despite observational studies consistently confirming lower serum 25(OH)D concentrations in patients with T2D or metabolic syndrome (4), most MR studies have not supported these conclusions (117). In a small subgroup of individuals with obesity and prediabetes, supplementation provided some modest benefit, albeit lower than lifestyle modifications or metformin (157). Of note, daily vitamin D supplementation (4000 IU) in the large D2d trial did not retard the progression of prediabetes into T2D. A post hoc and meta-analysis, however, suggested a possible beneficial effect in individuals with vitamin D deficiency (<30 nmol/L) at baseline or in participants who were able to achieve consistently high (≥100 nmol/L) serum 25(OH)D levels (152). Furthermore, analysis of the combined results of the D2d (US), Tromsø (Norway), and DPVD (Japan) RCTs—which were specifically designed and conducted to test whether vitamin D reduces the risk of diabetes in adults with prediabetes—showed that vitamin D supplementation reduced the risk of developing T2D in people with prediabetes not selected for vitamin D deficiency (158). In all 3 trials, the risk for diabetes was reduced in the group assigned to vitamin D compared to the placebo group, which did so in a remarkably similar way. The observed differences missed statistical significance in any trial because the reported risk reductions were smaller than each trial was powered to detect. An updated individual participant data meta-analysis of the same trials (159) showed that vitamin D reduced the risk of progression from prediabetes to diabetes by 15%. Also, vitamin D increased the likelihood of regression to normal glucose regulation by 30%, with no evidence of risk. In additional analyses, participants in the vitamin D group who maintained intratrial blood 25(OH)D of 50 ng/mL or greater ( ≥125 nmol/L) had a 76% risk reduction in new-onset diabetes compared to those who maintained blood 25(OH)D of 20 to 29 ng/mL (50-75 nmol/L). All participants received and were encouraged to follow the current lifestyle-based advice for diabetes prevention. Based on the results of this meta-analysis, the benefit-to-risk ratio of vitamin D to lower the risk of developing T2D in adults with prediabetes is favorable. These results should not be extrapolated to the general population at low or average risk for diabetes, as the benefit-to-risk ratio of high doses for diabetes prevention may not be favorable. Despite these promising results, some questions remain, that is, the optimal vitamin D dose or formulation and the specific blood 25(OH)D level to maximize benefit with little or no risk of any side effects (159).

Thus, the evidence from large-scale MR studies and RCTs is convergent and does not support vitamin D supplementation to prevent T2D in the general population. However, vitamin D supplementation benefits those with prediabetes and a predisposition to T2D, especially those with vitamin D deficiency. Additional studies or more in-depth analyses of the existing studies are needed to validate these findings (117, 159).

#### Mortality

Observational data have repeatedly linked poor vitamin D status with increased mortality. Large, older meta-analyses dealing mostly with women older than 70 years (160, 161) showed a 6% to 11% reduction in mortality. However, adding the newest megatrials eliminated this effect, possibly because they recruited a younger population. In these megatrials, overall mortality was much lower than shown in the previous meta-analyses (160, 161), and no effect of vitamin D supplementation on overall mortality was observed (128).

A Cochrane meta-analysis of 56 randomized trials including almost 100 000 participants, of whom were women older than 70 years, revealed that vitamin D, administered over 4 years, decreased mortality; this effect was seen in 38 trials of vitamin D_3_, but not with other forms of vitamin D (161). A newer meta-analysis of 52 RCTs, including more than 75 000 individuals, concluded that vitamin D (either vitamin D_3_ or D_2_) supplementation did not change mortality compared with no supplementation (162). Again, subanalyses found that vitamin D_3_ (instead of D_2_) supplementation tended to reduce mortality. Some MR studies found a link between lower predicted serum 25(OH)D and mortality, especially in individuals with rather poor vitamin D status (<16 ng/mL) (163-165). An individual participant data meta-analysis of almost 27 000 study participants with 25(OH)D levels standardized per VDSP protocols showed an association between low 25(OH)D and increased risk of all-cause mortality (166). The positive but small effect of vitamin D on mortality was confirmed by a recent umbrella review of observational, randomized, and MR studies (167). In conclusion, if vitamin D supplementation benefits extraskeletal health outcomes and major diseases, it is likely to have some effects on mortality, especially in older adults with poor vitamin D status, but not in younger, replete individuals (117).

### Summary of Vitamin D Deficiency-associated Clinical Outcomes

The long-known skeletal benefits of vitamin D and calcium related to rickets or osteoporosis remain valid. Most reported extraskeletal benefits of vitamin D were not confirmed by recent, large RCTs (see Table 3). The gradual increase in vitamin D levels in Western populations may explain these null findings, and older trials and meta-analyses may be more likely to show benefits because individuals were more likely to be vitamin D deficient than they are nowadays. RCTs and metanalyses published to date do not have adequate power to evaluate important subgroups, such as individuals with low 25(OH)D levels, men, the very old, ethnic groups other than White individuals, and those from low-income countries. Moreover, most of the studies use adverse events data to identify fractures and were performed in adults who were vitamin D replete at baseline in whom benefit would be unlikely and toxicity possible. Such studies confound the identification of possible beneficial effects in vitamin D–deficient individuals who might benefit from supplementation. Thus, when it comes to vitamin D, it is advisable to “giveth to those who needeth” (168). In fact, the benefit-to-risk ratio for vitamin D depends on the target population and medical condition. It would be incorrect to extrapolate vitamin D guidelines that apply to the general population (such as those from the US National Academic of Medicine) to avoid vitamin D deficiency (ie, rickets, osteomalacia) and promote bone health to special populations for whom the benefit-to-risk ratio of vitamin D would be different.

Nonetheless, RCTs, MR studies, and metanalyses suggest a link between vitamin D status with the immune system and diabetes, as well as fleeting effects on some CV events and some benefits on mortality risk when vitamin D_3_ is used.

## Vitamin D Supplementation

### Dosing Regimens

The term “*dose*” in relation to vitamin D is typically used to signify the measured quantity of vitamin D (usually cholecalciferol, but other formulations such as ergocalciferol, eldecalcitol, calcifediol, etc are also available) in a pill. It is expressed as µg or IU (where 10 µg is 400 IU). The dose of cholecalciferol is considered an important measure as it correlates with the change in blood 25(OH)D level, which is commonly used to define vitamin D status and correlates with important clinical outcomes. Doses can be considered as “loading” or “maintenance.” The most common use of a loading dose is to rapidly improve a low blood 25(OH)D; however, the clinical wisdom of this approach is questionable, especially given studies that demonstrate adverse effects with very high doses given infrequently, as discussed next. Intermittent administration of large doses is also used to optimize adherence. Daily doses are generally preferred when vitamin D replacement is considered necessary. The effect of a given dose on changing blood 25(OH)D varies considerably from person to person due to many factors, such as body weight, absorption, diet, degree of adiposity, CYP2R1 activity, DBP. The recommended dietary allowance for vitamin D by the National Academy of Medicine is set at 400 to 800 IU per day, and the tolerable upper intake level at 4000 IU per day; however, the “optimal” dose of vitamin D varies by the desired outcome, and other authors suggest that the upper limit of safety may be lower than 4000 IU per day (169-171). For example, 400 to 800 IU of vitamin D per day may be adequate to avoid clinical vitamin D deficiency and maintain calcium homeostasis in healthy individuals. Doses of vitamin D higher than the recommended upper limit may be associated with toxicity; nonetheless, daily doses up to 10 000 IU have been used without safety concerns (172). Careful and judicious use of vitamin D will permit the realization of potential benefits and achieving optimal outcomes.

Generally, there is a lack of consensus about the recommended vitamin D supplementation regimen (doses, administration schedule, treatment duration, etc) (173). Such heterogeneity can be explained, at least partly, by the scarcity of comparative pharmacokinetics studies for different dosing schedules (174-176). Moreover, different underlying conditions (eg, obesity) might reduce the effect of vitamin D supplementation (177, 178). Growing evidence suggests that the treatment schedule itself (ie, bolus vs frequent administration) may differently affect the effectiveness of the treatment (27, 179) and also clinical outcomes, with recent studies and a few meta-analyses showing more promising results with frequent administration schedules on skeletal and extraskeletal outcomes (4, 141, 144, 180, 181).

In this perspective, vitamin D supplementation guidelines should be specific for age group, body weight, ethnicity (skin type), and latitude of residence. For example, differences in serum 25(OH)D by BMI and absolute body weight have been reported (182-185). Vitamin D dose per kilogram of body weight per day could explain a 34.5% variation in circulating 25(OH)D in multivariable regression analyses of data pooled from several studies (184), leading to pronounced differences across BMI categories. Obese and overweight individuals tend to have serum 25(OH)D levels that are, on average, around 20 nmol/L lower and 8 nmol/L lower than those of normal-weight individuals, requiring 2.6 and 1.47 times higher supplementation, respectively (185). This is somewhat consistent with ES guidelines suggesting that the vitamin D dosage for obese people is “three times” greater than the recommended dose for individuals with normal body weight (46).

Another example of targeted, specific vitamin D dosing, of course, is in the pediatric setting. Infants and children have different upper tolerance limits compared to adults. To maintain a desirable 25(OH)D concentration, the 2010 IOM guidelines recommend 600 IU/d (15 μg) for children, adolescents, and adults, and 400 IU/d (10 μg) for infants (47). ES guidelines recommend 400 to 1000 IU/day (10-25 μg) for infants aged up to 1 year and 600 to 1000 IU/day (15-25 μg) for children older than 1 year to treat and prevent vitamin D deficiency (46). These values are consistent with several guidelines issued by other societies in the past several years. Of course, they can be increased if a laboratory-confirmed vitamin D deficiency is being treated (186).

Many studies investigated dosing regimens in pediatric patients. One trial comparing 4 different daily dosages (400, 800, 1200, 1600 IU) found that all dosages established 25(OH)D concentrations of 50 nmol/L or greater in 97% to 98% of infants at age 3 and 12 months, but only a dosage of 1600 IU/d 25(OH)D levels to 75 nmol/L or greater in 97.5% of infants at age 3 months; nonetheless, this study was discontinued prematurely because of elevated plasma 25(OH)D concentrations that have been associated with hypercalcemia (187). Another study also found that 1600 IU/day given for 10 weeks to infants from 2 weeks to 3 months of age maintained a 25(OH)D concentration above 80 nmol/L, but without causing hypercalcemia or hypercalciuria (188).

#### Daily supplementation

From a physiological perspective, daily administration of cholecalciferol seems to be most natural. Indeed, it appears that a daily approach results in higher efficacy in terms of 25(OH)D exposure and extraskeletal benefits.

In a recent RCT comparing 3 different dosing regimens in vitamin D–deficient participants with similar total end-of-study cumulative doses (D_3_ daily 10 000 IU 8 weeks, then 1000 IU for 4 weeks; 50 000 IU weekly for 12 weeks; and 100 000 IU every 2 weeks for 12 weeks), the group receiving the daily supplementation was the quickest to reach sufficiency (<2 weeks, although receiving a higher cumulative dose in the first 8 weeks when compared to the other 2 arms) and reached the highest serum 25(OH)D levels (172). Importantly, daily administration was associated with higher systemic exposure to 25(OH)D (greater area under the curve, +23% and +27% compared to weekly and biweekly administration, respectively), even when corrected for the cumulative dose (172). The greater 25(OH)D exposure of daily regimens could be due to lower activation of the 24-hydroxylase enzyme (CYP24A1). In an RCT of lactating women comparing the effect of bolus (150 000 IU) vs daily vitamin D_3_ dosing (5000 IU) on vitamin D_3_ catabolism, a single high-bolus dose of vitamin D led to greater production of 24,25(OH)_2_D_3_, relative to the 25(OH)D_3_ value than did daily vitamin D supplementation, with this effect persisting for at least 28 days after supplementation (27). The greater therapeutic potential of daily regimens compared to other regimens might be less relevant at lower doses (≤2000 IU). Two studies comparing 2000 IU/day vs 50 000 IU/month (189) and 800 IU/day vs 5600 IU/month (190) found no statistically significant differences in the 2 areas under the curves.

Greater 25(OH)D exposure and lesser 24-hydroxylase activity might be the rationale behind the potential extraskeletal benefits of cholecalciferol supplementation. The already cited metanalysis of 12 RCTs by Keum et al (141) found that the reduction in cancer mortality after vitamin D supplementation was largely attributable to interventions with daily dosing (as opposed to infrequent bolus dosing). Secondary analyses of the VITAL trial giving 2000 IU/day of cholecalciferol found a significant reduction in advanced cancers (metastatic or fatal), especially among those with normal BMI (126). However, the opposite was seen with monthly dosing in the D-Health trial, where the risk of death from cancer was increased (130). In the AMATERASU trial on patients with digestive-tract cancers, 2000 IU/day of cholecalciferol provided a cumulative hazard ratio of relapse or death of 0.66, significantly lower than the placebo group when adjusted by age quartile (191). Regarding the prevention of autoimmune diseases, supplementation with 2000 IU/day of cholecalciferol for 5 years reduced autoimmune diseases by 22% in the VITAL trial (129). Finally, a meta-analysis on the prevention of acute respiratory infections after vitamin D supplementation found that vitamin D given daily had an odds ratio (OR) of 0.78, compared to an OR of 0.97 to 0.98 if weekly or bolus regimens (145). Protection was mainly associated with administering daily doses of 400 to 1000 IU for up to 12 months and an age of 1.00 to 15.99 years at enrollment. This result is particularly interesting as recommended prevention strategies such as inactivated influenza vaccines in health adults probably reduce acute respiratory infections from 21.5% to 18.1% with a relative risk of 0.84 (192).

In conclusion, daily cholecalciferol administration might be the most efficient and beneficial strategy to increase serum 25(OH)D, at least from the biomedical (but not necessarily bio-psycho-social) perspective. Indeed, most RCT data suggesting extraskeletal benefits of cholecalciferol supplementation come from studies with daily dosing. Future studies should investigate this observation in pathologic conditions (ie, obesity).

#### Nondaily supplementation

Intermittent vitamin D dosing usually uses a greater amount to reach equivalent doses with fewer administrations. The rationale of this approach is to enhance adherence and ease management of specific patient groups, such as children and community-dwelling older people (193). Indeed, low adherence to vitamin D prescription has often been reported, although the topic is controversial. For example, Albrecht et al (194) recently investigated adherence to bone health–promoting lifestyle recommendations concerning osteoporosis status in a cross-sectional database of community-dwelling older adults (aged 65-75 years). In high-risk osteoporosis patients, adherence to vitamin D intake, defined as regular consumption of vitamin D–rich foods and/or vitamin D supplements, was high, ranging from 85% (women) to 93% (men). In contrast, in a cross-sectional study of pediatric outpatients affected by various diseases, Arshad et al (195) found that adherence to vitamin D prescription was quite low, particularly in those with diseases where vitamin D deficiency presents as a high-risk condition.

For these reasons, recurrent and protracted intervals of vitamin D supplementation appear to be an effective and convenient way to achieve and maintain sufficient vitamin D status and to increase patients’ adherence, but there is no agreement that treatment simplification with intermittent dosing significantly improves compliance (196) and there is consistent evidence to discourage the use of “megadoses” due to the possible side effects (197).

#### Weekly and monthly regimens

With equivalent doses and large formulations, daily, weekly, and monthly supplementation may lead to similar increases and levels of 25(OH)D in middle-aged (198) and obese individuals (199), in older individuals with hip fractures (200), and children with CKD (201). However, one study concluded that a daily regimen was more efficient in circulating 25(OH)D than weekly or monthly administration, but with different formulations (179). As compared with a daily regimen, a bolus dose is associated with a higher 24,25(OH)_2_D level and a higher 24,25(OH)_2_D to 25(OH)D ratio (27). In a monocentric, open-label randomized study in postmenopausal women, weekly vitamin D was more efficient than monthly in improving muscular function (measured through the Sit-to-Stand and Timed-Up-and-Go tests) (202). Monthly regimens have been tested in several large trials with multiple outcomes. Compared to a placebo, 100 000 IU monthly did not influence the risk of CVD, falls, fracture, or cancer, and lung or arterial functions in vitamin D–replete individuals (203). In those participants with baseline 25(OH)D lower than 50 nmol/L, the 100 000 IU vitamin D regimen increased lumbar spine BMD by 2.6% and improved lung and arterial functions (203). In the D-Health trial including more than 21 000 individuals, with 24% of them having a 25(OH)D level less than 50 nmol/L, 60 000 IU monthly did not influence all-cause mortality (126) but was associated with a higher risk of falls in those with a BMI of less than 25 (64). This observation was in agreement with another trial in which a higher percentage of fallers was detected with 60 000 IU/month compared to 24 000 IU/month over 1 year (204). In small trials, few episodes of hypercalcemia were reported with weekly doses between 20 000 and 100 000 IU in various target populations (205). Overall, trials with weekly or monthly vitamin D supplementation regimens did not show significant effects on clinical variables. This could be due to the recruited population (vitamin D–replete or obese individuals) or too large vitamin D doses leading to a U-shape dose-response relationship. Currently, there is no evidence of a superiority in the benefit/risk ratio of weekly or monthly vitamin D regimens over daily supplementation.

#### Longer intervals

Although one study using high doses with prolonged intervals (100 000 IU every 4 months) administered to community-dwelling adults older than 50 years found a reduction in fractures (206), other similar studies (500 000 IU every year (193)/150 000 IU every 3 months (207)) did not show a reduction in hip/vertebral/nonvertebral/total fracture incidence. This was also evidenced by the systematic review and meta-analysis of Zhao et al (208). In studies on the efficacy of vitamin D administration, the basal values of 25(OH)D are often either not measured (206) or are at normal/high levels (206), making it difficult to understand the real effect of supplementation on 25(OH)D values. In a subgroup analysis of Zhao's study, no differences in fracture incidence were found between intermittent high doses given once every year and other interval regimens (208). In Zhao's meta-analysis, reference is made to the study by Witham and colleagues (209) in which no negative effects of longer intervals of high-dose vitamin D administration on blood pressure in older patients with isolated systolic hypertension were reported.

Regarding the relation between long-term intervals of vitamin D administration and CVD risk, falls, and fracture outcomes in older and community-dwelling people, in a systematic review with meta-analysis, Barbarawi et al (142) did not find significant results favoring vitamin D intervention (100 000 IU every 4 months (189)/500000 IU yearly (197)) in preventing falls, fractures, or CVDs. Even in works cited in this meta-analysis, the basal 25(OH)D values were either not reported or sufficient.

In a systematic review with meta-analysis, Yang et al (210) cited 2 works that investigated the effect of intermittent high doses of vitamin D as adjuvant treatment in pneumonia in children (100 000 IU every 3 months (211) and 300 000 IU quarterly for 1 year (212)) on the incidence rate of repeated episodes of pneumonia, rate of intensive care unit (ICU) hospital admission, and complications rate. In both cases, no significant definitively positive effects were found. Regarding the safety of longer-interval vitamin D supplementation, in a recent systematic review with meta-analysis on children, Brustad et al (213) did not find any association with severe side effects. This was also seen in other studies with protracted intervals of vitamin D administration (211, 212, 214, 215).

### Summary of Vitamin D Dosing Regimens

In conclusion, one of the major justifications for longer intervals with high doses in vitamin D administration, namely, to address low compliance with more frequent regimens, is controversial. The rationale gains support in children and adolescents rather than in older individuals. However, it has to be taken into account that the cited meta-analyses underscored the point that there is no evidence of efficacy in intermittent high-dose and longer intervals of vitamin D administration in reducing fracture rate, falls, CV events, or infectious diseases. An increase in falls in older individuals has been observed with large, intermittent dosing (197, 216) (the literature regarding falls is somewhat controversial in part because there are no reliable methods to capture falls, as both diaries and self-reports are flawed). These conclusions should be tempered by inherent flaws in many reports in which the baseline vitamin D dosage or pretreatment 25(OH)D levels are not provided.

### Routes of Administration

Oral supplementation of cholecalciferol is the most commonly used approach. It is effective, simple, and generally safe. Therefore, it is the preferred way to supplement vitamin D. However, sometimes, the parenteral route may be a better method for improving vitamin D status than oral administration of vitamin D, particularly in situations like intestinal malabsorption. Interestingly, a new transdermal route of vitamin D administration is being proposed (217) but will not be discussed here due to the paucity of data.

#### Oral administration

Cholecalciferol (vitamin D_3_) and ergocalciferol (vitamin D_2_) are fat-soluble vitamins that are absorbed in the small intestine. Because they are lipophilic compounds, their absorption is similar to the absorption of lipids. Vitamin D is incorporated into micelles with biliary salts on the micelle surface. On average, about 80% of vitamin D is absorbed, but the variation in absorption can be large (55%-99%) (218-220). Taking vitamin D supplements with a fat-containing meal may improve vitamin D absorption (218, 220). Cholecalciferol and ergocalciferol are both rapidly absorbed, and the plasma levels peak after about 24 hours of ingestion. Absorption into the enterocytes of the intestinal wall was thought to be a passive process, but there is some evidence that vitamin D, especially in dietary doses, is also actively transported through the enterocyte membranes via cholesterol transporter proteins. However, passive transport seems to occur with pharmacological doses of vitamin D. From the enterocytes, vitamin D is exported in chylomicrons by the lymphatic route (218-220).

Bariatric surgery and intestinal malabsorption syndromes that reduce fat absorption, such as inflammatory bowel diseases, cystic fibrosis, and severe cholestasis, can also reduce vitamin D absorption (10, 221). However, intestinal malabsorption does not seem to affect the absorption of calcidiol as much, most likely because calcidiol is more water soluble, thus not requiring bile salts for absorption, and because calcidiol is absorbed by the portal route instead of the lymphatic route (219).

As cholesterol transporters are involved in vitamin D absorption, factors that interfere with cholesterol absorption could also affect vitamin D absorption. However, ezetimibe, an inhibitor of cholesterol transport, does not seem to affect vitamin D absorption despite the reduction in cholesterol absorption. There is also no strong evidence that phytosterols, plant sterols used to inhibit cholesterol absorption, impair vitamin D absorption. In contrast, there is some evidence that drugs used to reduce intestinal fat absorption, such as orlistat and olestra, may also reduce vitamin D absorption (219).

Vitamin D supplements are available in different vehicles, such as oil-containing gel capsules, oily drops, and hard powder tablets. Although it could be hypothesized that vitamin D would be better absorbed from oil-based vehicles, no convincing evidence supports this premise. In fact, there is some evidence that vitamin D may be better absorbed from a powder-based vehicle than from an oil-based vehicle in cases of intestinal fat malabsorption, such as in cystic fibrosis (222).

#### Parenteral administration

The optimal treatment of hypovitaminosis D in the general population and disease states is still debated (7). Parenteral administration of intermittent vitamin D boluses may be indicated in patients with hypovitaminosis D who are not suitable for oral intake or with intestinal malabsorptive diseases, including inflammatory bowel disease, celiac disease, pancreatic insufficiency, short-bowel syndrome, and post bariatric surgery (10, 221). Based on advantageous pharmacokinetic properties and evidence-based clinical data, intramuscular cholecalciferol may be the preferred form of vitamin D to be used in these clinical settings. In fact, it has been shown that cholecalciferol was able to reach higher serum 25(OH)D levels more rapidly than ergocalciferol when both vitamin D forms were administered as a single large intramuscular dose (300 000 UI) in adult or older patients with hypovitaminosis D (223-225). Moreover, in the study by Romagnoli et al (224), 2 months after administration of this large, intramuscular cholecalciferol dose, serum 25(OH)D levels were higher than those obtained after the same oral dose. Therefore, intermittent intramuscular cholecalciferol could be useful in clinical conditions when rapid correction of hypovitaminosis D is unnecessary and for long-term maintenance of adequate serum vitamin D levels, as in some older patients, to improve their adherence to vitamin D supplementation. However, safety concerns limit the clinical use of intermittent, excessive vitamin D doses. In fact, large intramuscular boluses (300 000 IU) induce unwanted effects such as an increase in falls and fracture events or enhance bone turnover (226, 227). There is a consensus to administer vitamin D boluses not higher than 100 000 IU (228). In conclusion, the therapeutic regimen to recover from vitamin D deficiency should be tailored to patients’ characteristics, such as age, BMI, severity of vitamin D deficiency, concurrent comorbidity, and use of other drugs.

### Different Forms of Vitamin D Supplementation

The main supplemental oral forms of vitamin D are cholecalciferol (vitamin D_3_) and ergocalciferol (vitamin D_2_). Both are readily available without a prescription. Cholecalciferol is the most used form of supplemental vitamin D. Calcidiol (calcifediol, 25(OH)D), the inactive vitamin D metabolite produced in the liver, and other vitamin D analogues, such as calcitriol (1,25(OH)_2_D, the physiologically active form of vitamin D) and alfacalcidol (1-hydroxyvitamin D), are used as prescription medicines in some conditions (Table 4).

**Table 4.** Characteristics of different forms/metabolites of vitamin D and when to use them

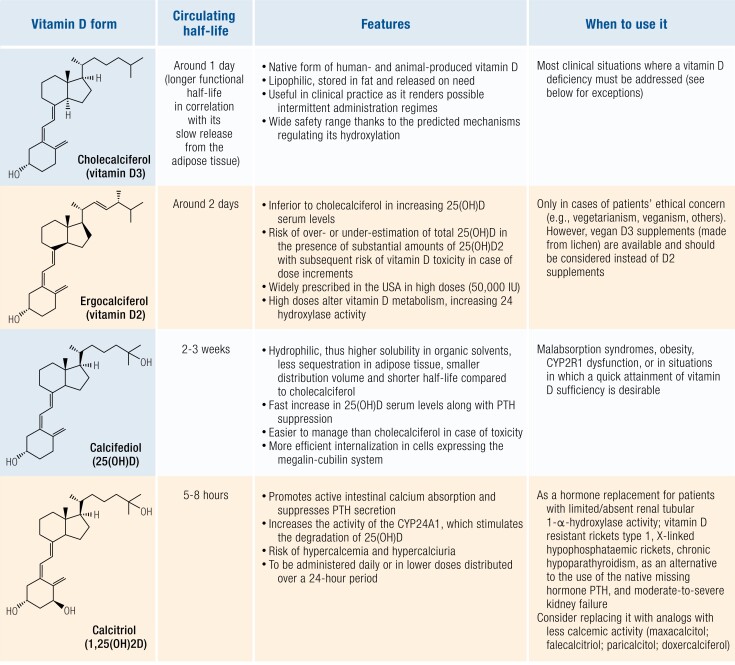

#### Ergocalciferol

Ergocalciferol does exist in nature (mainly in plants and fungi), and low circulating levels of 25(OH)D_2_ are present in free-ranging nonhuman primates and human population studies (229, 230). The 2 forms of vitamin D, cholecalciferol (D_3_) and ergocalciferol (D_2_), are often used interchangeably as supplementation or treatment of vitamin D deficiency as, historically, vitamins D_2_ and D_3_ were considered equally effective in treating rickets (231). Similarly, previous recommendations consider vitamins D_2_ and D_3_ interchangeable (46). Subsequently, however, multiple studies and meta-analyses comparing the effect of D_2_ and D_3_ on circulating 25(OH)D concentration have found cholecalciferol to be superior (223-225, 232).

Challenges to 25(OH)D measurement are widely recognized. The presence of 2 circulating 25(OH)D forms, 25(OH)D_3_ and 25(OH)D_2_, adds additional challenges, notably for automated immunoassays. Importantly, it is possible that the antibodies used in immunoassays may not detect 25(OH)D_2_ and 25(OH)D_3_ equally, and the proprietary approach to releasing 25(OH)D from DBP may not liberate the 2 forms equally (233). As such, it is perhaps unsurprising that multiple reports find overestimation or underestimation of total 25(OH)D in the presence of substantial amounts of 25(OH)D_2_ (233-236). This problem was corroborated by a recent interlaboratory comparison performed at the behest of the VDSP (87). Assay underestimation of total 25(OH)D in the presence of substantial amounts of 25(OH)D_2_ can have clinical consequences. A recent small report of patients receiving 50 000 IU of ergocalciferol every 2 weeks found 40% (6/15) to have total 25(OH)D levels less than 30 ng/mL when measured by immunoassay whereas all had values above 42 ng/mL when measured by LC-MS/MS (237). It is easy to imagine that such patients with “low” 25(OH)D values would have their dose increased, with at least potential toxicity, or undergo additional evaluation, such as for malabsorption. Thus, these assay issues are not clinically inconsequential.

Issues surrounding ergocalciferol use are of note for the United States, where 50 000 IU of vitamin D_2_ was the only high-dose preparation available by physician prescription and, therefore, ergocalciferol was widely prescribed. Now, instead, 50 000 IU of vitamin D_3_ is available by prescription. In addition to assay issues, widespread use of intermittent high-dose ergocalciferol (“bolus” therapy) appears to alter vitamin D metabolism, with increased 24-hydroxylase activity (27). Other adverse consequences of high-dose vitamin D therapy, notably increased fall risk (197), are reported and have led to calls to critically assess daily vs bolus vitamin D therapy (238).

To summarize, vitamins D_2_ and D_3_ are not equivalent in raising circulating 25(OH)D, and bolus dosing may have adverse effects on vitamin D metabolism and clinical outcomes. As such, it is to be expected that calls for the use of only cholecalciferol and avoidance of ergocalciferol have been and continue to be published (233, 239, 240) with recent osteoporosis-treatment guidance advising cholecalciferol over ergocalciferol (241) (see Table 4). Despite these recommendations, high-dose ergocalciferol remains widely prescribed in the United States.

#### Calcifediol

Calcifediol is the intermediate metabolite between cholecalciferol and calcitriol. Several pharmacokinetic studies performed in the last 4 decades have demonstrated its hydrophilic properties, leading to higher solubility in organic solvents, less sequestration in adipose tissue, smaller distribution volume, and shorter half-life when compared to cholecalciferol (242-244). By virtue of its hydrophilic properties, calcifediol is readily absorbed via the venous portal system and thus quickly increases circulating concentrations of 25(OH)D_3_. In contrast to cholecalciferol, which is mostly stored in fat tissue, 25(OH)D tends to be more evenly distributed throughout the body (20% in muscle, 30% in circulation, 35% in fat, and 15% elsewhere) (245). The administered dose will generally lead to predictable 25(OH)D levels and effective PTH suppression. In cases of toxicity, this form of vitamin D is easier to manage than cholecalciferol (244). Moreover, the greater affinity of calcifediol for DBP allows for more efficient internalization in cells expressing the megalin-cubilin system of endocytic receptors, such as the parathyroids and the renal tissue (246).

Such properties provide the rationale for using calcifediol in specific clinical conditions. The clinical situations that make use of calcifediol attractive are obesity, hepatic failure, patients with inactivating mutations of genes encoding CYP2R1 (the principal enzyme that is responsible for vitamin D 25-hydroxylation), or patients taking drugs that could influence the activity of cytochrome enzymes (ie, antiretroviral or antitubercular). Calcifediol was shown to have the same bioavailability in healthy adults with differing BMI and adults with intestinal malabsorption compared to controls (247). In an RCT on vitamin D–deficient, postmenopausal women, weekly calcifediol was found to be more effective and faster acting compared to cholecalciferol in increasing 25(OH)D serum levels. This more favorable kinetics led to greater improvement in muscle function (202). In another RCT in 35 healthy women aged 50 to 70 years, calcifediol given daily, weekly, or as a single bolus was about 2 to 3 times more potent in increasing plasma 25(OH)D_3_ concentrations than cholecalciferol (190).

New extended-release calcifediol formulations are more effective than cholecalciferol in raising serum 25(OH)D levels even in overweight nondialytic CKD patients with secondary hyperparathyroidism (248); nonetheless, it must be noted that these data arise from observational, retrospective data and subgroup post hoc analysis of RCTs.

Recently, retrospective studies have highlighted the role of calcifediol administration on various end points related to COVID-19 infection. To demonstrate a causative effect, Nogues et al (249) investigated 2 cohorts of patients with COVID-19, 1 of whom was untreated and 1 assigned to the oral calcifediol group. The treatment regimen consisted of oral calcifediol (0.532 mg the day of admission), followed by doses of 0.266 mg on days 3, 7, 15, and 30. Out of 447 patients treated with calcifediol at admission, 20 (4.5%) required the ICU, and 21 (4.7%) died; this was significantly lower compared to the untreated group of 391 patients, of whom 82 (21%) required the ICU and 62 (15.9%) died (both *P* ≤ .01). Adjusted logistic regression of calcifediol treatment on ICU admission indicates that patients treated with calcifediol had a lower risk of ICU admission (OR 0.02; 95% CI, 0.07-0.23) and mortality (OR 0.21; 95% CI, 0.10-0.43), suggesting an effectiveness of calcifediol treatment (249).

In summary, calcifediol seems to represent a form of vitamin D that is useful for replenishing vitamin D status. Most attractive clinical settings include malabsorption syndromes, obesity, CYP2R1 dysfunction, or situations in which quick attainment of vitamin D sufficiency is desirable (see Table 4).

#### Calcitriol

Calcitriol is the active hormonal form of vitamin D and the natural VDR ligand. It promotes active intestinal calcium absorption and suppresses PTH secretion. Calcitriol has a short half-life of around 5 to 8 hours; therefore, it should be administered daily (or with intermittent regimens) and sometimes in lower doses distributed over a 24-hour period (240, 250). As calcitriol is not an organic micronutrient, its use in clinical practice requires careful monitoring. Calcitriol increases the activity of CYP24A1, which stimulates the degradation of 25(OH)D. This results in serum 25(OH)D not being useful as a marker of adequate vitamin D supplementation and reduced potential benefits of physiological extrarenal/local production of calcitriol due to reduced substrate availability. Moreover, some studies have reported a more significant incidence of adverse events such as hypercalcemia and hypercalciuria. Thus, there is a need to monitor serum and urine calcium and phosphate (240, 251, 252). Because of these safety and clinical practicality issues, there is consensus that calcitriol use should be limited to hormone replacement for patients with limited/absent renal tubular 1-α-hydroxylase activity, as their capacity to produce calcitriol is severely limited (240, 251, 252). Indeed, calcitriol was first used to treat patients with vitamin D–resistant rickets type 1 (23). Other indications are X-linked hypophosphatemic rickets, chronic hypoparathyroidism, as an alternative to the use of the native missing hormone PTH, and moderate-to-severe kidney failure when calcitriol production is impaired or to suppress excessive PTH secretion. This use helps to control secondary hyperparathyroidism and resultant metabolic bone diseases. However, as calcitriol use is associated with frequent hypercalcemia, its use could be replaced by analogues with less calcemic activity approved for use in patients with secondary hyperparathyroidism in renal failure, in particular maxacalcitol (22-oxa-1,25(OH)_2_D_3_) and falecalcitriol (1,25(OH)_2_-26,27-F6-D_3_), which are currently available in Japan, and paricalcitol (19-nor-1,25(OH)_2_D_2_) and doxercalciferol (1α(OH)D2), available in the United States (253, 254). Calcitriol has also been proposed for the treatment of osteoporosis, but it is not approved in this setting (240, 251, 252).

In conclusion, calcitriol is not suitable for supplementation or nutritional fortification, and none of many excellent reviews, guidelines, and policy papers consider the use of calcitriol in the nutritional context (supplementation and fortification). However, guidelines suggest that vitamin D supplementation is advised in patients with chronic hypoparathyroidism, chronic kidney failure, and low vitamin D status in addition to receiving therapeutic doses of calcitriol (see Table 4). Such a recommendation is motivated by the activity of extrarenal 1-α-hydroxylase, which is compromised by reduced renal function (ie, not regulated by PTH) and is not regulated by feedback mechanisms (240, 251, 252).

### Vitamin D Safety and Monitoring

Vitamin D supplementation is generally a safe treatment with minimal adverse events and no need for strict monitoring. However, side effects of vitamin D treatment exist and can result in vitamin D toxicity (VDT).

#### Vitamin D toxicity

VDT is a clinical condition characterized by excess vitamin D (hypervitaminosis D), resulting in severe hypercalcemia that may persist for a prolonged period of time, leading to serious health consequences. Signs and symptoms of VDT are related primarily to hypercalcemia, with complications encompassing adverse events in the CV, renal, gastrointestinal, neurological, and musculoskeletal systems (255, 256). VDT prevalence is unknown, but it is rare due to the wide therapeutic index of vitamin D (255, 256). Evidence from systematic studies of VDT in humans is missing for ethical reasons, and data mostly stem from studies of VDT in animals and anecdotal reports. The condition of infantile hypercalcemia was first described in the United Kingdom and Switzerland, showing symptoms such as failure to thrive, osteosclerosis, developmental delay, and even death, but was not immediately associated with vitamin D intake. Suggestions were made that excess vitamin D intake may be a causative factor (children received up to 35 000 IU daily). Eventually, the British Ministry of Health suggested a substantial reduction in vitamin D allowance, resulting in a marked decrease in infantile hypercalcemia cases (257-260). As the prescriptions of vitamin D products are increasing worldwide, so is the number of VDT reports, with more than 75% published since 2010. Many of these cases result from inappropriate prescribing; moreover, the prescription of high-dose unlicensed and poorly manufactured treatments can be greater than 60%, as they are cheaper (261, 262).

In healthy individuals, hypervitaminosis D is usually defined as “exogenous” as it develops after uncontrolled use of megadoses of vitamin D or its metabolites or analogues, as in case of high dose of calcifediol leading to a faster increase in 25(OH)D serum levels compared with cholecalciferol but easier to manage than cholecalciferol in case of toxicity for its hydrophilicity and lesser sequestration in adipose tissue. On the other hand, excessive production of calcitriol in granulomatous disorders, lymphomas, primary hyperparathyroidism, and idiopathic infantile hypercalcemia results in “endogenous” hypervitaminosis D (255, 256).

VDT is defined by a biochemical phenotype with markedly elevated calcifediol concentrations (>150 ng/mL or >375 nmol/L), along with dihydroxylated metabolites (24,25(OH)_2_D_3_; 25,26(OH)_2_D_3_, 25(OH)D_3_-26,23-lactone), unless the causal agents are vitamin D analogues, such as paricalcitol. Calcitriol levels may be in the normal reference range or even reduced in exogenous VDT while elevated in endogenous VDT. PTH levels can be very low or undetectable (263). VDT thus results in severe hypercalcemia, hypercalciuria, and hyperphosphatemia. Pathogenetically, hypercalcemia is a consequence of high calcifediol levels in exogenous VDT (with calcifediol at pharmacological concentrations overcoming VDR affinity disadvantages and/or displacing 1,25(OH)D_2_ from DBP (264)), while high calcitriol levels cause endogenous VDT. Exogenous factors that interact with VDT risk include dosage, calcium in the diet or as a supplement, vitamin D intake with the diet, social status (ie, neglected patients), artificial UV light treatment sessions, quantity of supplement use, and time of exposure. Endogenous risk factors comprise age, sex, vitamin D status, hypersensitivity syndromes, and the pharmacogenetics of the vitamin D response and metabolism (253, 254, 263). This is why there is no clear cutoff above which VDT occurs and below which it does not.

In conclusion, VDT is a rare but life-threatening event mostly caused by unintentional overdosing due to pharmaceutical products. The prescriber and dispenser should avoid unlicensed vitamin D products. VDT should always be considered a differential diagnosis when evaluating patients with hypercalcemia. Future studies should encompass the evaluation of concurrent conditions that increase the risk of VDT and include the evaluation of classic and nonclassic adverse events for VDT.

#### Monitoring vitamin D status during treatment

Monitoring treatments is important to assess their efficacy and safety. Regarding vitamin D supplementations, there is limited evidence for when to monitor response to therapy or toxicity.

When it comes to achieving sufficiency in deficient patients, it seems there is no need to monitor differently according to different dosage regimens (dose and/or frequency) or baseline 25(OH)D serum values. The increase in serum 25(OH)D concentration after supplementation follows a curvilinear response with the increase of the cumulative doses (265, 266). The delta increase over 100 IU depends on baseline levels, and there is less increase per 100 IU with high doses than low doses (267). Van Groningen et al (268) calculated that the cholecalciferol loading dose required to reach the serum 25(OH)D target level of 75 nmol/L can be calculated as dose (IU) = 40 × [75 − serum 25(OH)D] × body weight. Mean 25(OH)D levels over a 2-month period are similar to daily, weekly, or monthly administrations (although monthly dosing is associated with more variability), and sufficiency can be reached independently from the baseline 25(OH)D values (200). In the study by Fassio et al (172), all participants normalized 25(OH)D safely, regardless of dosing regimens and including patients receiving 10 000 IU/day for the first 8 weeks; moreover, no cases of hypercalcemia were recorded. With regard to recent megatrials results, no effects were found on serum calcium or calciuria unless very high doses were used, such as 4000 to 10 000 IU per day in the Calgary study (104). Furthermore, these studies did not confirm the modestly increased risk of kidney stones observed in the WHI trial (400 IU per day) (117). However, there might be a need for monitoring in case of other vitamin D metabolite use. As discussed earlier, calcifediol acts much more rapidly than cholecalciferol in increasing serum 25(OH)D levels, resulting in greater fluctuation of 25(OH)D levels. For example, supplementation with 20 μg (800 IU) of cholecalciferol (vitamin D_3_) increases 25(OH)D concentrations toward 70 nmol/L (28 ng/mL) within 16 weeks, while supplementation with 10 or 15 μg calcifediol (25(OH)D) increases 25(OH)D levels more than 75 nmol/L (>30 ng/mL) in 8 and 4 weeks, respectively (269).

To summarize, cholecalciferol can maintain physiological 25(OH)D serum levels above 30 ng/mL (75 nmol/L) but below 50 ng/mL (125 nmol/L) for a long time, regardless of whether the dosage given is daily or intermittent (weekly, fortnightly, or monthly), due to its slow pharmacokinetic elimination caused by prolonged storage and release on demand according to physiological needs (270). Routine monitoring of 25(OH)D levels is generally unnecessary for patients on long-term maintenance vitamin D doses of up to at least 2000 IU/day. Retesting after 8 to 12 weeks from the start of supplementation may be appropriate when poor compliance is suspected, in case of symptoms suggestive of vitamin D deficiency, and for patients at risk of persistent 25(OH)D level below 30 ng/mL (75 nmol/L). These comprise institutionalized or hospitalized individuals, people in whom vitamin D therapy uncovers subclinical primary hyperparathyroidism, obese individuals, individuals undergoing bariatric surgery, individuals who use of certain concomitant medications (eg, anticonvulsant medications, glucocorticoids), and patients with malabsorption, including inflammatory bowel disease and celiac disease. For patients on potent antiresorptive agents (eg, denosumab or zoledronic acid), vitamin D levels should be checked annually per protocol (71).

## Conclusions

The metabolism, mechanisms of action, and pathophysiology of vitamin D and its multifaceted implications in human health have been extensively investigated for more than a century. However, the role of vitamin D status assessment and the detailed outcomes of vitamin D deficiency and its supplementation are still not completely understood. Thus, we extensively reviewed the literature on controversial vitamin D topics to better clarify and summarize the “whys, whens, and hows” of vitamin D assessment and supplementation in generally healthy populations and clinical conditions.

Vitamin D metabolism involves a different extensive panel of enzymes, resulting in various hormonal metabolites. Moreover, the VDR has been demonstrated to act as a key role transcription factor in most cells and can regulate a plethora of genes. New insights into the regulation of vitamin D–related enzymes and the differential mechanism of action of VDR have demonstrated potential links between several metabolic disorders and vitamin D effects. In this perspective, assessing a distinctive pattern of noncanonical vitamin D metabolites may allow us to better characterize different pathological conditions related to vitamin D metabolism that do not depend only on reduced solar exposure or vitamin D diet intake.

Besides the potential utility of the evaluation of noncanonical vitamin D metabolites, 25(OH)D is nowadays the most widely accepted biomarker to evaluate vitamin D status; however, its optimal levels are still debated. Recommendations on optimal 25(OH)D levels deriving from international societies and guidelines can differ due to the different approaches used, including clinical perspectives (level of cutoff at which no individual has an undesirable outcome) or public health perspectives (level of cutoff at which 97.5% of individuals do not have an undesirable outcome). Another critical issue is the lack of an accepted laboratory test assay standardization, and this prevents a proper interpretation of data reported by different studies, resulting in enabling rational data pooling and implementation of meta-analyses focused on vitamin D influence in various clinical outcomes of interest. Thus, 25(OH)D laboratory assays should be monitored in their performance through external quality assessment plans providing target reference values from standardized measurement procedures.

Vitamin D deficiency has been extensively related to the occurrence of skeletal disorders, such as rickets and osteomalacia. It can also be negatively implicated in osteopenia and osteoporosis, which must be mandatory and managed with vitamin D supplements. More recently, the interest in the putative extraskeletal effects of vitamin D have resulted in several clinical trials addressing vitamin D's influence on cancer and CV risk, respiratory effects, autoimmune diseases, diabetes, and mortality. The null results of some of these RCTs—especially the megatrials—hampered the enthusiasm around these topics. However, these trials were progressively revised, and their null results were mainly related to the enrollment of vitamin D–replete adults in whom benefit would be unlikely and the inhomogeneous methodologies in vitamin D supplementation with different forms, metabolites, and doses. Indeed, subsequent secondary analyses have progressively shown that vitamin D might be useful in reducing cancer incidence and mortality in the long term, in reducing autoimmune diseases and CV events (in particular central arterial hypertension, myocardial infarction, and atrial fibrillation) occurrence, and the development of diabetes from prediabetes forms. Nonetheless, these RCTs and the following meta-analyses were not powerful enough to evaluate these crucial subgroups, and further studies with better methodological conductions are warranted.

Regarding the different forms and metabolites used for vitamin D supplementation, oral administration is the preferred route, and parenteral administration should be reserved for special clinical situations, such as in patients with severe gastrointestinal malabsorption syndromes or after bariatric surgery. Cholecalciferol remains the preferred choice, and it is generally safe, requiring less strict monitoring. Ergocalciferol has been demonstrated to be less effective in raising 25(OH)D serum levels and, thus, should be reserved for specific clinical conditions. Calcifediol could be recommended in patients with obesity, malabsorption syndromes, CYP2R1 dysfunction, or in situations in which a quick, rapid achievement of vitamin D sufficiency is desirable. Calcitriol use should be limited for patients with limited/absent renal tubular 1-α-hydroxylase activity and in vitamin D–resistant rickets type 1, X-linked hypophosphatemic rickets, and chronic hypoparathyroidism.

Growing preclinical and clinical observations associating vitamin D with many health clinical conditions have been progressively reported in recent years. However, the lack of rigorous methodologies on patient enrollment, vitamin D supplements, and standardized laboratory assays have limited the ability to draw definitive conclusions about these data that still need to be more clearly understood.

Thus, a “whys, whens, and hows” of vitamin D assessment and supplementation derived from an international expert panel discussion about controversial topics regarding vitamin D metabolism, assessment, actions, and supplementation is needed to help the scientific community in evaluating and conducting future further studies with more rigorous methodologies, to better explore any clinical setting potentially influenced by vitamin D, and to provide reliable data required to update our international recommendations.

## Abbreviations

1,25(OH)_2_D: 1,25 dihydroxyvitamin D
7-DHC: 7-dehydrocholesterol
25(OH)D: 25-hydroxyvitamin D
BMD: bone mineral density
BMI: body mass index
CDC: Centers for Disease Control and Prevention
CKD: chronic kidney disease
CVD: cardiovascular disease
DBP: vitamin D binding protein
ES: Endocrine Society
HAT: histone acetyltransferase activity
HDAC: histone deacetylase activity
ICU: intensive care unit
IL: interleukin
IOM: Institute of Medicine
LC-MS: liquid chromatography–mass spectrometry
miRNA: microRNA
MR: mendelian randomization
OR: odds ratio
PTH: parathyroid hormone
RCT: randomized controlled trial
SRC: steroid hormone receptor coactivator
T2D: type 2 diabetes
TSS: transcription start site
UVB: sunlight
VDR: vitamin D receptor
VDSP: Vitamin D Standardization Program
vitamin D_2_: ergocalciferol
vitamin D_3_: cholecalciferol
VDT: vitamin D toxicity
